# Premalignant SOX2 overexpression in the fallopian tubes of ovarian cancer patients: Discovery and validation studies

**DOI:** 10.1016/j.ebiom.2016.06.048

**Published:** 2016-07-02

**Authors:** Karin Hellner, Fabrizio Miranda, Donatien Fotso Chedom, Sandra Herrero-Gonzalez, Daniel M. Hayden, Rick Tearle, Mara Artibani, Mohammad KaramiNejadRanjbar, Ruth Williams, Kezia Gaitskell, Samar Elorbany, Ruoyan Xu, Alex Laios, Petronela Buiga, Karim Ahmed, Sunanda Dhar, Rebecca Yu Zhang, Leticia Campo, Kevin A. Myers, María Lozano, María Ruiz-Miró, Sónia Gatius, Alba Mota, Gema Moreno-Bueno, Xavier Matias-Guiu, Javier Benítez, Lorna Witty, Gil McVean, Simon Leedham, Ian Tomlinson, Radoje Drmanac, Jean-Baptiste Cazier, Robert Klein, Kevin Dunne, Robert C. Bast, Stephen H. Kennedy, Bassim Hassan, Stefano Lise, María José Garcia, Brock A. Peters, Christopher Yau, Tatjana Sauka-Spengler, Ahmed Ashour Ahmed

**Affiliations:** a Ovarian Cancer Cell Laboratory, Weatherall Institute of Molecular Medicine, University of Oxford, Headington, Oxford OX3 9DS, UK; b Nuffield Department of Obstetrics and Gynaecology, University of Oxford, Women's Centre, John Radcliffe Hospital, Oxford OX3 9DU, UK; c Complete Genomics, Inc., 2071 Stierlin Ct., Mountain View, CA 94043, United States; d University of Oxford, Gene Regulatory Networks in Development and Disease Laboratory, Weatherall Institute of Molecular Medicine, Radcliffe Department of Medicine, Oxford OX3 9DS, UK; e Trinity College, University of Cambridge, Cambridge CB2 1TQ, UK; f Department of Histopathology, Oxford University Hospitals, Oxford OX3 9DU, UK; g Department of Oncology, University of Oxford, Old Road Campus Research Building, Roosevelt Drive, Oxford OX3 7DQ, UK; h Histopathology Core Unit, Spanish National Cancer Research Centre (CNIO), Madrid, Spain; i Biobank, Institut de Recerca Biomèdica Lleida (IRBLLEIDA), Lleida, Spain; j Department of Pathology and Molecular Genetics, Hospital Universitari Arnau de Vilanova, University of Lleida, IRBLLEIDA, Lleida, Spain; k Department of Biochemistry, Universidad Autonoma de Madrid (UAM), Instituto de Investigaciones Biomédicas “Alberto Sols” (CSIC-UAM), IdiPAZ, Madrid, Spain; l MD Anderson international Foundation, Madrid, Spain; m Human Genetics Group, Spanish National Cancer Research Center (CNIO), Madrid, Spain; n Biomedical Network Research Centre on Rare Diseases (CIBERER), Spain; o Wellcome Trust Centre for Human Genetics, NIHR Biomedical Research Centre, Roosevelt Drive, Oxford OX3 7BN, UK; p BGI-Shenzhen, Shenzhen, China; q Centre for Computational Biology, University of Birmingham, Edgbaston B15 2TT, UK; r Department of Experimental Therapeutics, M.D. Anderson Cancer Center, University of Texas, USA; s Tumor Growth Control Group, Sir William Dunn School of Pathology, South Parks Road, Oxford OX1 3RE, UK; t Department of Statistics, 1 South Parks Road, Oxford OX1 3TG, UK

**Keywords:** Ovarian cancer, Fallopian tube, BRCA mutations, SOX2, Screening, Precancer

## Abstract

Current screening methods for ovarian cancer can only detect advanced disease. Earlier detection has proved difficult because the molecular precursors involved in the natural history of the disease are unknown. To identify early driver mutations in ovarian cancer cells, we used dense whole genome sequencing of micrometastases and microscopic residual disease collected at three time points over three years from a single patient during treatment for high-grade serous ovarian cancer (HGSOC). The functional and clinical significance of the identified mutations was examined using a combination of population-based whole genome sequencing, targeted deep sequencing, multi-center analysis of protein expression, loss of function experiments in an in-vivo reporter assay and mammalian models, and gain of function experiments in primary cultured fallopian tube epithelial (FTE) cells. We identified frequent mutations involving a 40 kb distal repressor region for the key stem cell differentiation gene *SOX2*. In the apparently normal FTE, the region was also mutated. This was associated with a profound increase in SOX2 expression (*p* < 2^−16^), which was not found in patients without cancer (*n* = 108). Importantly, we show that SOX2 overexpression in FTE is nearly ubiquitous in patients with HGSOCs (*n* = 100), and common in BRCA1-BRCA2 mutation carriers (*n* = 71) who underwent prophylactic salpingo-oophorectomy. We propose that the finding of SOX2 overexpression in FTE could be exploited to develop biomarkers for detecting disease at a premalignant stage, which would reduce mortality from this devastating disease.

## Highlights

• Dense sequencing of an ovarian cancer identifies founder non-coding mutations.
• Fallopian tube SOX2 overexpression is a common premalignant feature in ovarian cancer.
• The expression of SOX2 and MYC in the fallopian tube appears mutually exclusive.

A major obstacle for effective early detection of ovarian cancer is that CA125 and ultrasound can only detect established ovarian cancer. Currently proposed pre-cancer lesions are not amenable to clinical detection because they affect only a small number of cells. In this work we demonstrated that pre-cancer SOX2 overexpression in the FTE is nearly ubiquitous in HGSOCs and is also a common feature in women with BRCA1 and BRCA2 mutations prior to ovarian cancer initiation. Thus, our data has important implications for screening, although it will need additional clinical evaluation to test the feasibility of quantitative detection of SOX2 expression or the expression of its downstream targets for this purpose.

## 1 Introduction

Because of late presentation and chemotherapy resistance ovarian cancer is the deadliest amongst all gynecological malignancies. Over 80% of ovarian tumors are high-grade serous ovarian cancers (HGSOCs), which is a particularly fatal type due to its initial asymptomatic but aggressive growth behavior. Current methods of detection have been successfully implemented for detection and possible reduction of mortality from ovarian cancer (Menon et al., 2015, Drescher et al., 2013). However, such methods are only capable of the detection of established invasive cancers. Understanding the natural history of the disease and the discovery of novel markers for detection at a premalignant stage will enable the effective control of ovarian cancer.

In this work, we prospectively analyzed the genomic composition of a single tumor over a three-year period to identify driver mutations that may have contributed to the initiation of the tumor. We identified non-coding mutations that cluster near genes involved in stem cell regulation. We established that one mutation is located in a previously unrecognized repressor element of *SOX2*, an important stem cell gene, and is associated with induction of SOX2 expression. We demonstrate that the expansion of SOX2-expressing cells within the fallopian tube epithelium is a common feature of HGSOCs, a crucial finding that opens new avenues for early disease detection prior to clinical presentation.

## 2 Materials and Methods

### 2.1 Overall Description of the Study Design

**Fig. 1:**
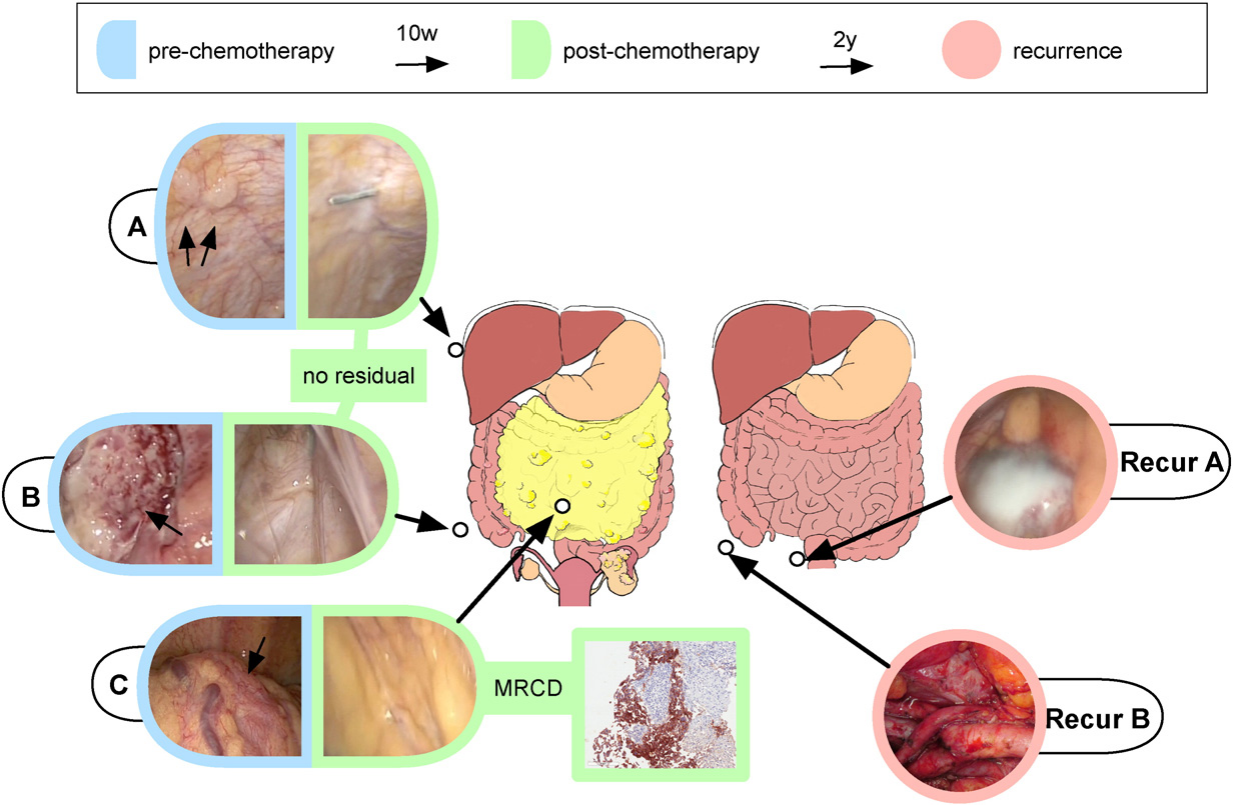
An ovarian cancer model for investigating primary chemotherapy resistance. A diagram illustrating the sites from which the biopsies were obtained in patient 11152 and the corresponding intra-operative images of the biopsy sites. The sub-diaphragmatic peritoneum (site A), the para-cecal peritoneum (site B) and the omentum (site C) were sampled in the primary tumor. A para-rectal mass (Recur A) and a pelvic node (Recur B) were sampled at presentation of disease recurrence. Note the complete macroscopic resolution of the tumor following chemotherapy (also see Supplementary video). TP53 immunohistochemical staining of a tumor islet from MRCD is also presented. A diagram illustrating the sites from which the biopsies were obtained in patient 11152 and the corresponding intra-operative images of the biopsy sites. The sub-diaphragmatic peritoneum (site A), the para-cecal peritoneum (site B) and the omentum (site C) were sampled in the primary tumor. A para-rectal mass (Recur A) and a pelvic node (Recur B) were sampled at presentation of disease recurrence. Note the complete macroscopic resolution of the tumor following chemotherapy (also see Supplementary video). TP53 immunohistochemical staining of a tumor islet from MRCD is also presented.

The clinical samples for this translational study were obtained from patients recruited to the Gynecological Oncology Targeted Therapy Study 01 (GO-Target-01) and the Oxford Ovarian Cancer Predict Chemotherapy Response Trial (OXO-PCR-01) under research ethics approval number 11-SC-0014 and 12-SC-0404, respectively. We performed intraoperative video recording to document sampling sites (Supplementary video). Strict standard operating procedures were used to diminish the risk of DNA cross-contamination during sample collection and processing. Whole genome sequencing (WGS) of laser capture microdissected tumor islets (*n* = 30, Supplementary Fig. S1A) and bulk tissue samples of a single HGSOC (patient study ID: 11152). We obtained WGS data from 39 samples from three independent data sets and a tumor recurrence set (Supplementary Fig. S1B). Samples were obtained from different locations before chemotherapy, after neoadjuvant chemotherapy and approximately two and half years later at the time of first recurrence (Fig. 1, Supplementary Table 1). Complete macroscopic clearance at all sites (Supplementary video) as well as microscopic clearance of the peritoneal implants at sites A and B were documented following chemotherapy. Microscopic residual chemoresistant disease (MRCD) was only detected at the omentum in position C. Sequencing of tumor islets was performed using long fragment read (LFR) WGS (Peters et al., 2012) of 30 tumor islets (typically comprising only tens of cancer cells). A table of variants was generated and filtered using “cgatools” and custom scripts. GREAT software (McLean et al., 2010) was used for gene ontology analysis. Targeted sequencing was carried out for validation of mutations using 100 bp paired-end sequencing and read mapping was performed using STAMPY software (Lunter and Goodson, 2011), whilst variants were identified using PLATYPUS (Rimmer et al., 2014). Digital PCR was performed using the Droplet Reader (BioRad) and analyzed with the QuantaLife software (BioRad). For the in-vivo studies, fertilized chicken eggs were electroporated with enhancer constructs and analyzed as previously described (Betancur et al., 2010, Simoes-Costa et al., 2012). Targeted deletion of the BB5 region was performed using Clustered Regularly Interspaced Short Palindromic Repeats (CRISPR) (Cong et al., 2013). Chromatin immunoprecipitation (ChIP) assays were carried out in primary fallopian tube epithelial cells (Cong et al., 2013) and a fraction of the products was used for either real-time PCR reactions or high throughput sequencing. Immunohistochemistry studies were carried out on the Leica Bond Max staining system including the appropriate positive and negative controls. Detailed materials and methods are available in the Supplementary methods section.

### 2.2 Translational Studies

#### 2.2.1 Ethical Approval

Tumor islets and blood samples for whole genome sequencing were obtained from case 11152 who provided written consent to participate in the prospective biomarker validation study Gynecological Oncology Targeted Therapy Study 01 (GO-Target-01) under research ethics approval number 11/SC/0014. Targeted sequencing was performed on samples from patients who participated in the same study and patients who participated in the prospective Oxford Ovarian Cancer Predict Chemotherapy Response Trial (OXO-PCR-01), under research ethics approval number 12/SC/0404. Necessary informed consents from study participants were obtained as appropriate.

#### 2.2.2 Clinical History and Sample Collection of Patient 11152

Patient 11152 presented with radiological evidence of at least stage IIIC ovarian cancer with evidence of multiple omental and peritoneal nodules, subcapsular splenic nodules and left paracardiac lymph node enlargement and elevated CA125 tumor marker. The patient had a diagnostic laparoscopy and biopsy to confirm the diagnosis of HGSOC. On the day of the procedure, tissue samples were retrieved, split in halves and either saved in cryovials and immersed in dry ice within minutes from obtaining the biopsy in theatre or formalin fixed for standard histological diagnosis. Research samples were stored in − 80 °C. In addition, a blood sample was obtained in theatre and saved in sterile EDTA-collection tubes. The patient received three cycles of paclitaxel and carboplatin and had near complete response. Following ten weeks from the first laparoscopy, the patient underwent a diagnostic laparoscopy to evaluate chemotherapy response, prior to proceeding, in the same session, to standard debulking surgery. Guided by the first video-laparoscopy, samples were obtained from the same sites from which the initial biopsies were taken.

#### 2.2.3 Precautions for Tissue Handling to Diminish the Risk of Cross Contamination of DNA

Samples were collected from − 80 °C to a box of dry ice and transported within the same building to the cryostat room. Individual cryovials were obtained from the dry-ice box and immediately placed within a pre-cooled cryostat stage at − 25 °C. The cryostat (CryoStar 70X, Thermo Scientific) was not previously used for routine diagnostic or research work. The cryostat stage and dissection board were cleaned by ethanol prior to use for individual samples, and gloves were changed before cutting each new tissue biopsy. For each sample, a single dissection blade (MB DynaSharp Microtome Blade, Thermo Scientific) and single sterile forceps (that was cleaned, individually wrapped and autoclaved prior to use) were used. Samples were obtained from the cryovials and placed on a sterile dish and cut using disposal single-use blades to obtain a piece of tissue for processing. These tissue pieces were then placed on individual new dissection discs for snap freezing in optimal cutting temperature (OCT) compound (NEG-50, Richard-Allan Scientific) prior to microtome cutting.

#### 2.2.4 Section Processing for Laser Capture Microdissection

The first tissue section was mounted onto regular glass slides (SuperFrost Plus, VWR International) for hematoxylin (Hematoxylin solution, Gill No. 3, Sigma) and eosin (Eosin Y solution, Sigma) staining (H&E), according to manufacturer's instructions, followed by six to ten sequential tissue sections at 6 μm thickness onto polyethylene naphthalate membrane (PEN) glass slides (MembraneSlide 1.0 PEN, Zeiss) that were pre-exposed to UV light for 30 min. Slides were immediately stored at − 80 °C. Nuclease-free technique was used throughout the procedure and buffers and alcohol solutions were cooled to 4 °C and used fresh each time. Each H&E slide was reviewed by a gynecological oncology pathologist (SD) to confirm the presence of cancer cells and delineate their location. PEN slides were dipped in 50% ethanol for fixation and rinsed in H_2_O to remove excess OCT compound. The slides were stained with cresyl violet (Sigma Aldrich) at a concentration of 0.1% (weight/volume) in 50% ethanol for 15 s, rinsed in 50% ethanol and immediately used for microdissection. For formalin-fixed, paraffin embedded (FFPE) material, 6 μm sections were cut onto activated PEN slides and dried at 56 °C overnight, then dewaxed in Xylene (Sigma) and rehydrated through graded alcohols to water, then briefly dipped in 1% methyl green (Sigma), washed in H_2_O. The slides were dried at 37 °C for 1 h and then used for microdissection. Laser capture microdissection was performed on a PALM Laser Microdissection System (Zeiss) and the cut tissue was catapulted into 200 μl membrane caps (AdhesiveCap 200 opaque, Zeiss, Jena, Germany) and immediately stored on dry ice. Images of empty caps as well as target area in 5 ×, 10 × and 20 × magnification were obtained prior to microdissection for documentation. To maintain DNA integrity, slides were kept on dry ice until microdissection and microdissection was performed for no longer than 45 min per slide. Caps were stored at − 80 °C until sequencing.

#### 2.2.5 Macrodissection of FTE

Serial unstained FFPE tissue sections (10 μm) were aligned with the corresponding H&E stained section in which the location of the FTE was pen marked. With a sterile, single-use scalpel blade tip the FTE tissue was scrapped off the glass slide and mounted onto 200 μl a membrane cap (AdhesiveCap 200 opaque, Zeiss, Jena, Germany). To avoid cross contamination, the slides and caps were placed in sterile tissue culture dishes. DNA extraction and amplification was performed using the REPLIg FFPE kit (Qiagen). For macrodissection of the p53 signature from FFPE immuno-stained sections the slides were incubated overnight in Xylene at 37 °C. The coverslip was lifted and the slide soaked in Xylene for 30 min, then washed in gradient ethanol. The tissue was macrodissected using a scalpel blade tip. DNA was extracted with the Arcturus® PicoPure® DNA isolation kit (Life technologies) and amplified using the REPLIg mini kit (Qiagen) according to manufacturer's instructions. DNA was quantified as described below.

### 2.3 DNA Extraction

For DNA extraction from frozen tissues 10 to 20 scrolls of tissue were cut at 60 μm thickness using a cryostat as described above. For FFPE samples, 10 to 20 scrolls of 20 μm thick sections were dewaxed with Xylene and washed with 100% ethanol. DNA was extracted using the DNeasy blood and tissue kit (Qiagen) according to manufacturers instructions. DNA concentrations were quantified using the PicoGreen (Quant-iT™ PicoGreen® dsDNA Assay Kit, Life technologies), A260/280 ratio and absorption spectra were generated using a spectrophotometer (Nanodrop ND-1000, Thermo Scientific) and broad range Qubit system (Invitrogen) and quality was checked using a 1% agarose E-gel (Invitrogen).

### 2.4 Sequencing

#### 2.4.1 Targeted Sequencing

LFR sequencing and whole genome sequencing were performed as previously described (Dean et al., 2002, Drmanac et al., 2010). A summary of the clinical characteristics of patients for whom targeted sequencing was performed is presented in Supplementary Table 2. For sequencing the 2 Mb flanking the *SOX2* gene, the online NimbleDesign tool (http://www.nimblegen.com/products/nimbledesign/index.html) was used to design capture primers (Roche). For sequencing of the test set the TruSeq® DNA HT Sample Preparation Kit (Illumina) was used to allow for multiplexing and captured using the Seq Cap EZ Choice Library (Roche). Both kits were used according to manufacturer's instructions. Library quality control was carried out using the broad range Qubit system (Invitrogen) and the 2200 TapeStation (Agilent). Sequencing was carried out on a HiSeq2500 (Illumina) using TruSeq® Rapid SBS 100 bp paired end sequencing. For sequencing the validation set of matched tumor/normal DNA pairs, a more focused library of capture primers was designed targeting ~ 1.6 Mb flanking SOX2 (chr3: 180,806,202–182,429,714). DNA from paired samples and ChIP products were fragmented (S2, Covaris) and sample libraries were constructed and multiplexed using Seq Cap EZ kit (Roche) according to manufacturer's instructions. Capture was performed as described above. Quality control was carried out on a 2100 Bioanalyzer (Agilent) and library concentrations were measured with the high sensitivity Qubit system (Invitrogen). Sequencing was performed on a MiSeq platform (Illumina) using v3 MiSeq chemistry. To increase coverage, the libraries for the blood sample and the microdissected tubal epithelium of case 11152 were recaptured and sequenced using v2 MiSeq chemistry, 100 bp paired end.

#### 2.4.2 Sanger Sequencing

For DNA sequencing using dye-terminator method, *TP53* exon 8 was amplified and sequenced using primers TP53-forward ‘GGGTGCAGTTATGCCTCAGATT’ and TP53-reverse ‘CGGCATTTTGAGTGTTAGACTGG’ as previously described (Ahmed et al., 2010). SOX2 BB5 was amplified and sequenced using the BB5-forward ‘CACCCATGTGAATCATCTCG’ and BB5-reverse ‘ACCAGGTGTCCGAGAGTACG’ primers. PCR was performed using the high fidelity DNA Phusion polymerase (NEB) as per manufacturer's instructions. Sequencing was performed for the rare variants identified in patients (Supplementary Table 3) using the primers listed in Supplementary Table 4.

#### 2.4.3 Digital Droplet PCR

Digital droplet polymerase chain reaction (PCR) was performed on duplicate samples. Primers 5833217_F; 5′-ACCTACTAGACCCCAGGCAAG-3′ and 5833217_R; 5′-GGCGCAGGAGGAGACC-3′ were used to amplify a 60 bp amplicon containing the BB5 nucleotide and either detected using 5833217_V; 5′-CCTGGGACCCAAACC-3′ VIC-labeled probe for wild type or 5833217_M; 5′-CTGGCACCCAAACC-3′ FAM-labeled probe for mutant amplicons (TaqMan® SNP Genotyping Assays, custom design, Roche Molecular Systems). *TP53* mutation was quantified using primers 22410689_F; 5′-CTGTGCGCCGGTCTCT-3′ and 22410689_R; 5′-TGGGACGGAACAGCTTTGAG-3′ to amplify a 64 bp amplicon and detected using 22410689_V 5′-TGCGTGTTTGTGCCTG-3′ VIC-tagged probe for wild type and 22410689_M; 5′-TGCGTGTTTTTGCCTG-3′ FAM-tagged probe for mutant amplicons. Reactions were prepared using droplet digital PCR Super Mix (BioRad) and standard PCR performed according to manufacturer's instructions. Amplification events were detected with a digital PCR plate reader (QX100 Droplet Reader, BioRad) and data was analyzed using the QuantaSoft Software (Version 1.3.2.0, BioRad). Average droplet count was 11,728 per sample. Samples with < 7000 droplets were excluded from the analysis.

### 2.5 Sequencing Analysis

#### 2.5.1 Whole Genome Sequencing

Reads were mapped to the reference genome (GRCh37) and variants were called by local de novo assembly as previously described (Carnevali et al., 2012). A table of variants across the various tumor and normal genome assemblies was generated and filtered using the Complete Genomics cgatools program and custom scripts. Sequence data were deposited in the European Genome-Phenome Archive (EGA) with the following accession number: EGAS00001001909.

#### 2.5.2 Performing Gene Ontology Enrichment Analysis

The functional prediction of *cis*-regulatory regions was performed using the Genomic Region Enrichment of Annotation Tool (GREAT) as previously described (McLean et al., 2010). In brief, GREAT assigns regulatory domains for each gene that consists of a basal domain (5 kb upstream and 1 kb downstream of transcription start site [TSS]) plus extension of up to 1 Mb, but not beyond 1 Mb, in both directions to the nearest gene basal domain. The enrichment for a particular biological process gene ontology (which consists of a number of genes) was computed by obtaining the ratio of the fraction of foreground (FG) variants (i.e. the ancestor variants) that mapped to genes of a gene ontology *x* to the fraction of background (BG) variants (e.g. progeny variants) that mapped to the same gene ontology. To be regarded as a gene ontology hit, we required that a gene ontology had a false discovery rate “Q value” of < 0.01 and a number of genes supporting a particular ontology of more than ten. The BG was defined as either the progeny variants (variants present in two or more tumor sites), the whole genome or the number of germline variants as indicated in the text. Permutation analysis was based on the assumption that random samples from the background of equal size to the FG should not give higher enrichment the one observed for the true FG. To test this, we obtained 10,000 samples of 750 variants from the BG and computed the enrichment for each and counted the number of times in which that enrichment was higher than the one observed by the true FG and presented the result (*p*-value) as the fraction of the count from the total number of random samples. The number of genes that GREAT assigned to the gene ontology “stem cell differentiation” was 73 and this was the basis of the analysis described above. We repeated the above analysis using the total number of human genes (283) assigned to the same gene ontology “GO:0048863” at the Gene Ontology data base (http://amigo.geneontology.org/cgi-bin/amigo/go.cgi) and by using a subset of genes (*n* = 81) that had an experimentally verified link to stem cell differentiation by selecting the human genes that satisfied any of the following terms: IDA; inferred from direct assay, IEP; inferred from expression pattern, IGI; inferred from genetic interactions, IMP; inferred from mutant phenotype.

#### 2.5.3 Analysis of Targeted Sequencing Data

Reads were mapped to the reference genome (GRCh37) using STAMPY software (Lunter and Goodson, 2011) and variants were identified using an in-house developed software, PLATYPUS (Rimmer et al., 2014). Downstream analysis was performed using in-house developed scripts. There were a total of 37,291 variants identified by sequencing. Further analysis focused on single nucleotide substitutions in cancer samples (*n* = 18,456 in 33 samples). To identify high quality rare variants, the analysis was restricted to variants that were present in less than five samples, were not called in the 1000 Genomes Project (Abecasis et al., 2012), and had a high quality score (i.e. flagged as “pass” or “allele bias” by PLATYPUS). Only 861 variants met these criteria and these are shown in Supplementary Table 5. In order to compute whether the observed rare variants displayed regional enrichment in the ovarian cancer set we adopted a simulation approach. First, we obtained minor allele frequencies (MAF) for common SNPs (defined as having MAF > 5%) from the 1000 Genomes Project in the 2 Mb region flanking SOX2. We then simulated genotypes, based on those minor allele frequencies, for samples consisting of 33 individuals (we ignore linkage effects) and counted the number of heterozygotes or non-reference homozygotes observed across the 33 simulated profiles. This process was repeated 1000 times in order to obtain an average profile of common SNP density across the region. We then examined 40 kb windows, spaced at overlapping intervals of 100 bp, and counted the number of rare variants observed across the 33 ovarian cancers. For each window, we then computed the test statistic (observed number of rare variants in ovarian cancers/expected number of common SNPs). In order to assess whether the calculated ratios were significantly enriched, we randomly sampled 100 subsets of 33 individuals from the wellderly (well elderly individuals) dataset and computed the same statistic for each subset by counting rare variants falling in any particular window. We then computed a *p*-value from the order-statistic of the observed test statistic in the ovarian cancers compared to those of the wellderly data.

In addition, the 861 variants were annotated using data from the Encyclopedia of DNA Elements (ENCODE) (Bernstein et al., 2012) to identify those that were within regulatory regions as reported by digital genomic footprint (Neph et al., 2012) downloaded from (ftp://ftp.ebi.ac.uk/pub/databases/ensembl/encode/integration_data_jan2011/byDataType/footprints//jan2011/), by DNAseI hypersensitivity (Thurman et al., 2012) downloaded from (http://hgdownload.cse.ucsc.edu/goldenPath/hg19/encodeDCC/wgEncodeRegDnaseClustered/) or by ChIP-Seq (Yip et al., 2012) experiments downloaded from (http://hgdownload.cse.ucsc.edu/goldenPath/hg19/encodeDCC/wgEncodeRegTfbsClustered/). In order to assess the degree of local enrichment of variants we first computed the global rate of variant occurrence over the 2 Mb region (113 variants/2,000,000 bp). We then considered a series of 40 kb overlapping windows spaced at 100 bp intervals spanning the 2 Mb. For each window, we counted the number of variants observed in the tumors and computed the probability of observing (at least) this many variants under the null hypothesis of uniform variant occurrence. We assumed that, under the null hypothesis, the number of observed variants follows a Poisson distribution with a rate parameter given by the global rate over the region.

#### 2.5.4 Analysis of ChIP-sequence Tracks

Following the ENCODE guidelines (Landt et al., 2012), read quality was first assessed using FastQC (v.0.10.1; http://www.bioinformatics.babraham.ac.uk). Low quality reads were filtered by using Trim galore (v 0.3.1; http://www.bioinformatics.babraham.ac.uk) for paired reads. The 3′ and 5′ adapters were trimmed using the Cutadapt program implemented in Trim galore. Only reads with a length > 60 bp after trimming were retained. Reads were then mapped against the human reference genome (build hg19) using bowtie (Langmead and Salzberg, 2012). Only non-duplicated aligned reads were retained. Multiple alignment of read was not allowed. To identify enriched regions, peaks were called on the immune-precipitated samples versus their input controls using Model-Based Analysis of ChIP-seq 2 (Feng et al., 2012) (MACS2 version 2.0.10) with a *p*-value threshold of 0.001 and default parameters. Signal track was built following the guideline at https://github.com/taoliu/MACS/wiki/Build-Signal-Track. Exploratory analysis and generation of figures was performed in R using custom scripts.

#### 2.5.5 Motif Analysis for the BB5 Variant

The motif UW.Motif.0169 overlapping the BB5 variant was discovered from digital footprints data. The logo plot in Supplementary Fig. S6A was generated using the webLogo3 software (http://weblogo.threeplusone.com/).

### 2.6 Fallopian Tubes Primary Epithelial Cell Culture

Patients scheduled to undergo surgical procedures provided written consent, prior to surgery, agreeing to participate in the study. The infundibular region of the fallopian tube was isolated, dissected and opened to reveal the lumen. Fallopian tubes were incubated in 15 ml conical tubes containing 0.5% trypsin and 0.1% DNaseII in MEM for 1 h at 37 °C, with shaking. The supernatant, containing the epithelial cells, was removed and mixed with 10% FBS in DMEM. Fallopian tube epithelial cells (FTEC) were centrifuged and plated in 10% DMEM. The purity of epithelial cells was checked by immunofluorescence. Optional purification using CD326 microbeads (Miltenyi Biotech) was if further purification was required.

### 2.7 Viral Transduction

pLx302-SOX2 was generated using Gateway system (Invitrogen) according to the manufacturer's instructions. *SOX2* was cloned into pDONOR221 (Invitrogen) from pMXs-Sox2 (Addgene) plasmid by BP Gateway clonase (Invitrogen) to generate entry clones. Using LR Gateway clonase (Invitrogen) SOX2 was cloned into the destination vector pLx302 (a gift from Dr. David Root (Yang et al., 2011); Addgene plasmid 25896). Packaging cells (HEK-293T) were co-transfected with the packaging vector p8.91, the envelope plasmid pMDG (gifts from Dr. Didier Trono, University of Geneva, Switzerland) and pLX302-SOX2 as previously described (Besnier et al., 2002). Following 72 h the HEK293-T medium containing the virus was collected, filtered through a 0.45 μm Minisart NML Syringe Filter (Sartorius) and stored at − 80 °C for later transduction.

### 2.8 Cloning, Mutant Generation and Chicken Embryo Transfection

The 1 kb regions flanking the BB5 SNP was cloned from Human genomic blood DNA by PCR amplification using Phusion high fidelity polymerase (NEB) according to the manufacturer's instructions using the following primers BB5_F; ‘TTTTTTCGTCTCgccaggTTACTCCAATATGAGAGATAAGAGCA’ and BB5_R; ‘TTTTTTCGTCTCcaacagCGCTCACACGGTGATTAGAA’, Test1_F; ‘TTTTTTCGTCTCgccaggCATTACTGGCAGCTGAGGGG’ and Test1_R; ‘TTTTTTCGTCTCcaacagTGATTTTCCCTGGGCAGACA’, Test2_F; ‘TTTTTTCGTCTCgccaggCTACTAGACCCCAGGCAAGG’ and Test2_R; ‘TTTTTTCGTCTCcaacagTGATTTTCCCTGGGCAGACA’, Test3_F; ‘TTTTTTCGTCTCgccaggTCCCTGTTCCTCACTCCTCT’ and Test3_R; ‘TTTTTTCGTCTCcaacagTGATTTTCCCTGGGCAGACA’. Products were cloned into a ptk citrine – *Bsm*BI vector. Restriction digestion of the PCR products and the vector was performed using *Bsm*BI enzyme (NEB) and the digested product was inserted into the vector using a T4 ligase (NEB). Point mutants were generated using QuikChange® II Site-Directed Mutagenesis Kit (Stratagene) as per manufacturer's instructions using the citrine vector containing each region and the primer sets SOX2_BB5mut_F; ‘ACCTGGGCCTGGCACCCAAACCCTT’ and SOX2_BB5mut_R ‘AAGGGTTTGGGTGCCAGGCCCAGGT’. DNA sequences of all cloned PCR products were verified with direct sequencing.

Fertilized chicken (*Gallus gallus*) eggs (Henry Stewart & Co. Ltd, Louth, UK) were incubated at 37 to 38 °C for approximately 20 to 24 h prior to electroporation. In the UK no license is required at the embryo stage used for this work. The entire epiblast of stage four chicken embryos (staged according to Hamburger and Hamilton (Hamburger and Hamilton, 1992)) was electroporated with enhancer constructs, cultured using modified new culture (Sauka-Spengler and Barembaum, 2008) and analyzed as previously described (Betancur et al., 2010, Simoes-Costa et al., 2012) (*n* = 10 to 15 per group). Neither randomization nor blinding was carried out. Non-vital or damaged embryos were excluded from further analysis. Immunofluorescence for detection of endogenous Sox2 expression and co-localization with citrine expression was carried out as previously described (Betancur et al., 2010, Simoes-Costa et al., 2012) using anti-Sox2 antibody (ab97959, Abcam) or anti-GFP antibody (MAB3580, Millipore) and detected using alexa-488 conjugated and alexa-568 conjugated secondary antibodies (Invitrogen) and observed using an inverted confocal microscope (LSA 510 META, Zeiss).

### 2.9 Chromatin Immunoprecipitation (ChIP) Assay

ChIP was performed on FTEC and HEK293T cells (purchased from ATCC, mycoplasma tested) using the commercially available ChIP-IT express enzymatic kit (Active Motif) according to manufacturer's instructions and the following antibodies: H3K27ac (C15410174 lot: A.7071-001P; Diagenode) and EZH2 (C15410039; lot: 003; Diagenode). Briefly, one million cells were cross-linked using formaldehyde 1% for 10 min and the reaction was stopped by adding glycine for 5 min at room temperature. Cells were then washed twice with ice cold PBS containing 1 × PMSF, and then resuspended in cell lysis buffer containing 1 × protease inhibitor cocktail and PMSF and rotated at 4 °C for 30 min. Nuclei were extracted using a Dounce homogenizer (pestle B) and treated with an enzymatic shearing cocktail at 37 °C for 20 min. Digested chromatin was immunoprecipitated at 4 °C overnight by adding protein G magnetic beads and rabbit IgG (cat. N. 12-370; lot. N. 1969696; Millipore) as a negative control. Following washes, reverse cross-linking and proteinase K treatment the DNA was purified using the Chromatin IP DNA purification kit (Active Motif). Sequencing of ChIP material was performed as described above. Real-time PCR was performed in a 96-well plate qPCR machine (ABI 7000, Applied Biosystems). Reactions were set up using SybrGreen (Applied Biosystems) according to the manufacturer's protocols using 1 μl of each ChIP reaction or 1: 100 to 200 dilution of the input fraction. The ΔΔCt method was used for quantification and calculations performed according to ChIP-qPCR data analysis instructions (Supper-Array, Bioscience Corporation). The following primers for the BB5 region were used: 5833217_F 5′-ACCTACTAGACCCCAGGCAAG-3′ and 5833217_R 5′-GGCGCAGGAGGAGACC-3′. For EZH2 inhibition, cell were treated for 96 h with 5 μM of UNC1999 (Sigma) before chromatin extraction.

### 2.10 CRISPR Vector Construction, Cell Culture and Transfection

Vector px330 was used as previously described (Cong et al., 2013). A pair of oligonucleotides (CRISPR-BB5-F; 5′-CACCGAGGGTTTGGGTCCCAGGCCC-3′ and CRISPR-BB5-R; 5′-AAACGGGCCTGGGACCCAAACCCTC-3′) encompassing the BB5 nucleotide (underlined) and extending up to a protospacer adjacent motif [AGG] (not shown) were annealed, phosphorylated, and ligated to the linearized vector.

HEK293 cells (ATCC) were maintained in DMEM (Invitrogen) supplemented with 10% fetal bovine serum and 100 U/ml penicillin/streptomycin and incubated at 37 °C and 5% CO_2_. HEK293 cells were transfected with the construct px330-BB5 using FUGENE HD (Promega) according to the manufacturer's instructions. After two weeks, the DNA for each clone was extracted using DNeasy blood and tissue kit (Qiagen) and the region flanking the BB5 nucleotide was amplified using the following pair of primers: SH-BB5-01-F; 5′-TCCAATATGAGAGATAAGAGCAC-3′ and SH-BB5-01-R; 5′-GCTGAAAAGACCAAACTTAAAAC-3′. HEK293T cells were transfected and two days later single cells were plated in 96-well plates and harvested for DNA collection on the indicated dates. The number of positive clones carrying bi-allelic mutations (nominated as homozygous), the number of clones carrying mono-allelic mutation (nominated as heterozygous) and clones with no deletions (nominated as negative) are reported in Supplementary Table 6.

### 2.11 Immunofluorescence

FTEC or SKOv3 cells were fixed with 4% (w/v) formaldehyde in PBS for 4 min and then permeabilized overnight with 100% ethanol at − 20 °C. Cells were then blocked with 1.5% BSA in TBS for 1 h and incubated with primary mouse antibody against pan-cytokeratin (ab7753, Abcam), primary rabbit antibody against MYC (9402S, Cell Signaling Technology), primary rabbit antibody against SOX2 (3579, Cell signaling Technology), primary mouse antibody against SOX2 (4900, Cell Signaling Technology) or primary rabbit antibody against PAX8 (10336, Proteintech) for 1 h. Cells were washed using washing buffer (TBS + Triton 0.2% + SDS 0.04%) and then incubated with donkey anti-mouse or donkey anti-rabbit conjugated with Alexa Fluor 488 or Alexa Fluor 546 secondary antibodies (Invitrogen), respectively. After three washes cells were mounted using DAPI-containing mounting media (Vector Laboratories).

### 2.12 Immunohistochemistry

A summary of patients from both groups (discovery set and validation sets) for whom SOX2 IHC was performed is presented in Supplementary Table 7. Power calculations were performed to determine the minimum specimen number for the validation set based on the data from the discovery set (mean % for benign: 3, s.d.: 4; mean % for HGSOCs: 14, s.d.: 10, difference between means: 11%) assuming a desire for 90% power and a 5% significance level. To identify the difference in 3 + staining intensity for the above scenario we estimated a group size of ten cases.

Tissue sections of 2.5 μm or 4 μm thickness were cut from FFPE tumor or control samples. Automated staining was carried out with the Leica Bond Max autostainer (Leica Microsystems) or Autostainer plus Link 48 (Dako®). In short, antigen retrieval at 100 °C for 20 min was followed by primary antibody incubation with the rabbit anti-SOX2 (3579, Cell Signaling Technology) or IgG control for up to 1 h then detection using the BOND™ Polymer Refine Detection System (DS9800, Leica Biosystems) as per manufacturer's instructions. Double staining was achieved using the BOND™ Polymer Refine Detection System and the BOND™ Polymer Refine Red Detection System (DS9390, Leica Biosystems) according to the manufacturer's instructions. Stained slides were scanned at 20 × and 40 × magnification on the Aperio slide scanner (Aperio) or Mirax scan (Zeiss) fitted with a 40 ×/0.95 Plan Apochromat objective lens (Zeiss) for the second validation set (CNIO; Spanish National Cancer Research Centre). The ImageScope software (v11.2.0.780, Aperio) was used for quantification of nuclear staining for the discovery set and the first validation set. For scoring of SOX2 positivity in fallopian tubes, only the tubal epithelium was marked by using the “negative pen tool” to exclude stroma. The marked FTE was analyzed with the program algorithm “nuclear v9” which scored the staining of all nuclei within the marked area and assigned scores of 0 for not detectable signal, + 1 for weak staining, + 2 for moderate staining and + 3 for strong staining. Nuclear positivity was confirmed on selected areas using the “deconvolution” algorithm to subtract SOX2 from underlying hematoxylin staining. The diagnosis and clinical details are provided in Supplementary Table 7. For scoring of SOX2 expression in HGSOCs tumor foci were marked and benign tissue excluded and the procedure completed as described above. For scoring the second validation set (CNIO), the AxioVision 4.6.3 software (Zeiss) pattern recognition training software was used. Algorithm parameters were set by using the AxioVision Wizard so that threshold and filter conditions (size, diameter and perimeter of different spots) were established to train the application in a noise reduction method that determines the different positive activation signals: 0 for no signal, + 1 for weak staining, + 2 for moderate staining and + 3 for strong staining. Immunohistochemistry for other proteins was conducted in a similar manner to the method described above using anti-p53 (Clone DO-7, Dako) and anti-WT1 (Clone WT1-562, Leica) antibodies, anti-TUBB4 (T7941, Sigma), anti-MYC (ab32072, Abcam), anti-EZH2 (Clone 6A10, Leica).

## 3 Results

### 3.1 Laparoscopy-guided Prospective Multi-region Sampling in an Ovarian Cancer Patient

We set out to discover early (root) driver mutations in a single high-grade serous ovarian tumor that may have contributed to its genesis. We hypothesized that such early mutations would be present in all cancer cells irrespective of their location, time of presentation or biological behavior (e.g. chemotherapy response). To account for these three variables, we utilized intraoperative video recording to document the site of sampling before and after chemotherapy and at recurrence and to obtain a direct visual evaluation of chemotherapy response. LFR WGS technology enabled the accurate sequencing of very low (picogram) quantities of DNA (Peters et al., 2015) which is not currently possible by standard WGS or other methods of long fragment sequencing (Kuleshov et al., 2014). Since post-chemotherapy samples only had microscopic residual disease, using this technique on laser-capture microdissected cancer islets was essential.

We identified 750 unique mutations that were present in practically all tumor islet samples in our discovery set (≥ 90% of tumor islets) irrespective of space, tumor site or biological behavior. These mutations were extensively confirmed using standard WGS and targeted sequencing (Supplementary Fig. S2A). The variants included two synonymous mutations, eight non-synonymous mutations in protein-coding regions, and 740 mutations in non-coding regions (Supplementary Table 8). One mutation, detected in exon 8 of *TP53*, was shared between all tumor islets but was not present in normal stroma samples from patient 11152. This result was consistent with the previous finding by Ahmed et al. (2010) and Anon (2011) of ubiquitous *TP53* mutations in HGSOCs and has, therefore, validated our approach of discovering potential driver mutations.

### 3.2 Non-coding Mutations Cluster at Potential *cis* Regulatory Elements of Genetic Drivers of Stem Cell Differentiation

We mapped the 750 mutations to the nearest transcription start sites of genes and performed pathway-enrichment analyses of the identified genes as previously described (McLean et al., 2010). This analysis revealed five significantly enriched ontologies of biological processes (Supplementary Fig. S2B, Supplementary Table 9, Supplementary Table 10) that were all related to embryonic and stem cell differentiation. Furthermore, the stem cell differentiation ontology was supported by 23 mutations that mapped to 15 genes (e.g., *SOX2*, *PAX7*, *WNT7A*). This finding was confirmed by several iterations of analysis (Supplementary Fig. S3) and multiple rounds of further sequencing (Supplementary Fig. S1A). The identification of *SOX2*, a key driver of stem cell differentiation (Takahashi and Yamanaka, 2006) that was recently implicated in skin cancer tumor initiation (Boumahdi et al., 2014), was particularly notable in our analysis because of the proximity of a total number of six mutations to the *SOX2* gene locus. We termed the nucleotides, in which the mutations mapping near SOX2 occurred, BB1 to BB6.

### 3.3 The BB5 Mutation Occurred at the Pre-neoplastic Lesion of the Tumor and Marked a Region That Was Frequently Mutated in HGSOCs

**Fig. 2:**
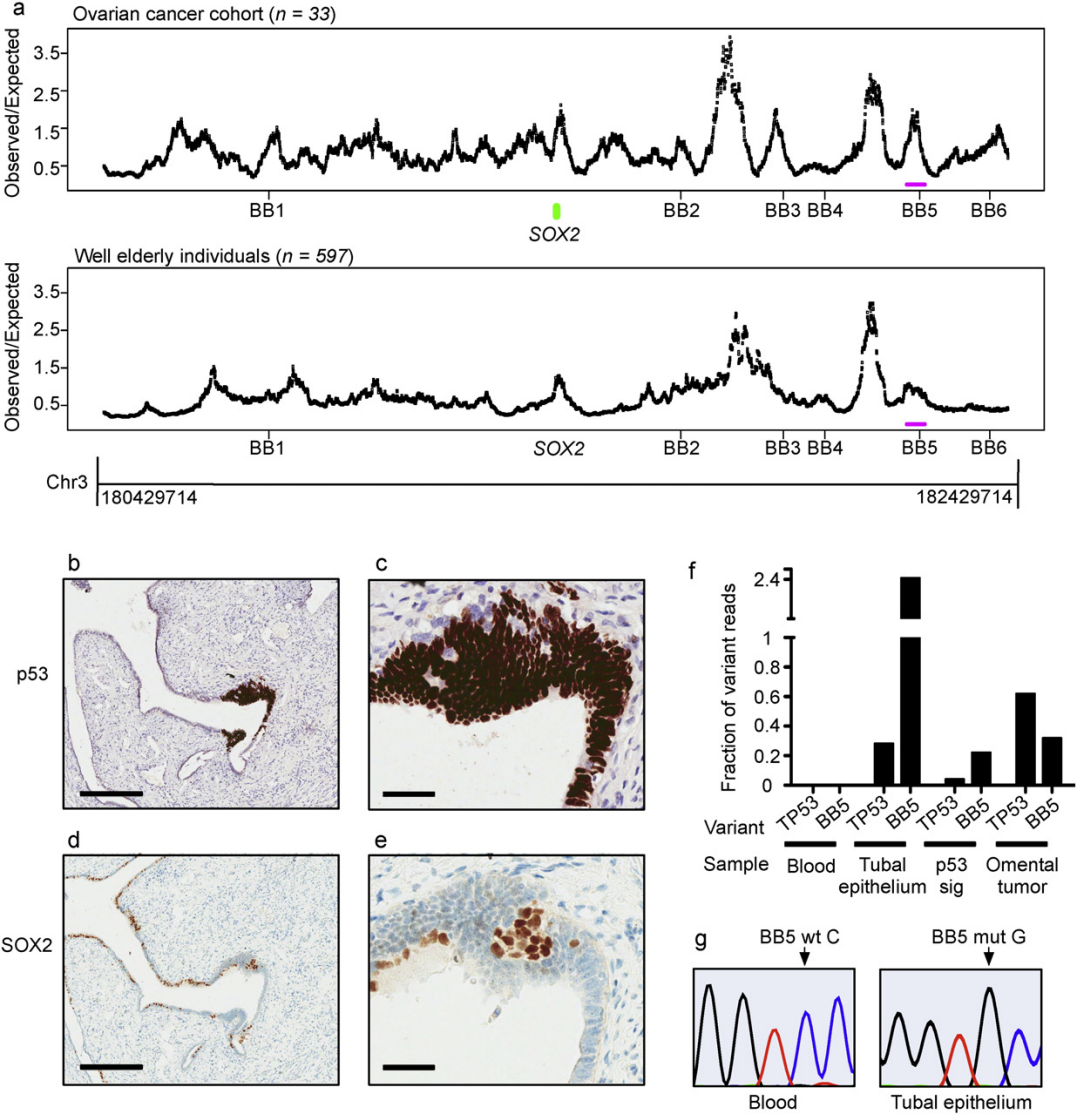
A 40 kb region is frequently mutated in HGSOCs and consists of a distal *SOX2* repressor. a. To determine whether any of the six mutations (BB1 to BB6) mapping near the SOX2 gene locus marked regions that were sites of frequent occurrence of private variants or mutations in HGSOCs, we performed deep-targeted sequencing of the 2 Mb region flanking *SOX2* on 33 HGSOCs (Supplementary Table 2). A total of 861 single nucleotide substitutions (Supplementary Table 5) were identified that were not previously reported in the 1000 Genomes Project (median = 21, range = 11 to 97). Because functionally important genomic regions tend to be significantly less susceptible to genomic variation within a population, we determined whether the identified rare variants accumulated in specific areas within the 2 Mb region that were less susceptible to genomic alterations on a population scale. To test this hypothesis, we constructed overlapping moving windows of 40 kb size and compared the observed frequency of rare mutations (not previously described in the 1000 Genomes Project) in our group of patients. The expected frequency of SNPs in the same windows was based on 1000 sets of simulated cohorts of 33 individuals from the previously reported 1000 Genomes Project data. Upper panel: shown is the ratio of the observed number of variants in 40 kb “moving” windows in the cancer set to the expected number of variants in the equivalent windows based on 1000 permutations of simulated 1000 Genomes Project data. Based on this analysis, a peak observed/expected ratio (enrichment statistic) was identified in a 40 kb region flanking the BB5 nucleotide referred to as the BB5 region. Lower panel: To test whether this observation was higher than what would be expected by chance, we sequenced germline DNA from 597 healthy elderly volunteers and sequenced germline DNA from 436 individuals from the 1000 Genomes Project at higher depth. We then identified rare variants in the elderly set and repeated the above analysis. Comparing the enrichment statistic in the BB5 region in the cancer set to that obtained from 100 permutations of 33 individuals from the elderly set confirmed the significant enrichment of rare variants in the ovarian cancer set (*p* < 0.01). b–e. Immunohistochemical staining of p53 and SOX2 at low (B and D, scale bar = 300 μm) and high (c and e, scale bar = 50 μm) magnification in normal FTE and the p53 signature in patient 11152. Note the strong focal p53 staining at the multi-layered epithelium (p53 signature). f. The fraction of mutant reads relative to the total number of reads of the BB5 and *TP53* mutations in germline DNA, the FTE, the p53 signature (p53 sig) and the tumor of the index case (*n* = 4 repeats). g. Sequencing trace indicating the BB5 mutation. a. To determine whether any of the six mutations (BB1 to BB6) mapping near the SOX2 gene locus marked regions that were sites of frequent occurrence of private variants or mutations in HGSOCs, we performed deep-targeted sequencing of the 2 Mb region flanking *SOX2* on 33 HGSOCs (Supplementary Table 2). A total of 861 single nucleotide substitutions (Supplementary Table 5) were identified that were not previously reported in the 1000 Genomes Project (median = 21, range = 11 to 97). Because functionally important genomic regions tend to be significantly less susceptible to genomic variation within a population, we determined whether the identified rare variants accumulated in specific areas within the 2 Mb region that were less susceptible to genomic alterations on a population scale. To test this hypothesis, we constructed overlapping moving windows of 40 kb size and compared the observed frequency of rare mutations (not previously described in the 1000 Genomes Project) in our group of patients. The expected frequency of SNPs in the same windows was based on 1000 sets of simulated cohorts of 33 individuals from the previously reported 1000 Genomes Project data. Upper panel: shown is the ratio of the observed number of variants in 40 kb “moving” windows in the cancer set to the expected number of variants in the equivalent windows based on 1000 permutations of simulated 1000 Genomes Project data. Based on this analysis, a peak observed/expected ratio (enrichment statistic) was identified in a 40 kb region flanking the BB5 nucleotide referred to as the BB5 region. Lower panel: To test whether this observation was higher than what would be expected by chance, we sequenced germline DNA from 597 healthy elderly volunteers and sequenced germline DNA from 436 individuals from the 1000 Genomes Project at higher depth. We then identified rare variants in the elderly set and repeated the above analysis. Comparing the enrichment statistic in the BB5 region in the cancer set to that obtained from 100 permutations of 33 individuals from the elderly set confirmed the significant enrichment of rare variants in the ovarian cancer set (*p* < 0.01). b–e. Immunohistochemical staining of p53 and SOX2 at low (B and D, scale bar = 300 μm) and high (c and e, scale bar = 50 μm) magnification in normal FTE and the p53 signature in patient 11152. Note the strong focal p53 staining at the multi-layered epithelium (p53 signature). f. The fraction of mutant reads relative to the total number of reads of the BB5 and *TP53* mutations in germline DNA, the FTE, the p53 signature (p53 sig) and the tumor of the index case (*n* = 4 repeats). g. Sequencing trace indicating the BB5 mutation.

We next performed deep-targeted sequencing of the 2 Mb region flanking the *SOX2* gene locus and BB1 to BB6 on tumor samples from 33 patients with HGSOCs, including case 11152 (Table 1, Supplementary Table 2), and identified 861 single nucleotide polymorphisms (SNPs) in this region (Supplementary Table 5) that were not previously reported in the 1000 Genomes Project (median = 21, range = 11 to 97 per cancer sample). Comparing the frequency of occurrence of these SNPs with those previously identified in the 1000 Genomes Project or with SNPs identified from whole genome sequencing analysis of a new set of 597 healthy elderly volunteers combined with deep whole genome re-sequencing of DNA from 436 individuals from the 1000 Genomes Project identified a 40 kb region flanking the BB5 nucleotide (Fig. 2A), that was significantly enriched in rare SNPs (*p* < 0.01, permutation analysis). These mutations significantly targeted biochemically active sites (implying that they were active regulatory elements) as determined by mining the ENCODE data for DNaseI hypersensitivity or transcription factor binding activity (Supplementary Fig. S4). We therefore chose to characterize this 40 kb region, referred to as the BB5 region, further.

Sequencing results from tumor and normal tissue revealed that the BB5 region (chr3: 182,189,714–182,229,714) included 21 single nucleotide substitutions in HGSOCs from 16 patients (48.5% of all patients), of which nine variants from seven patients (21.2%) were somatic mutations (Supplementary Table 3). Independent validation by deep-targeted sequencing of a 1.6 Mb region flanking SOX2 in tumor-normal tissue pairs of 16 additional patients with HGSOCs identified two further mutations in the BB5 region in two patients.

Examining the fallopian tube as a potential tissue of origin of HGSOCs (Crum, 2009, Lee et al., 2007, Kurman and Shih Ie, 2011, Karst et al., 2011) led to the identification of the p53 signature (Lee et al., 2007), indicated by p53 nuclear overexpression in fallopian tube epithelium (FTE), in patient 11152 (Fig. 2b–c). A subpopulation of the cells at the signature also expressed high levels of nuclear SOX2 (Fig. 2d–e). Using a combination of deep-targeted sequencing, droplet-digital PCR and Sanger sequencing, we confirmed that both, the BB5 nucleotide and *TP53*, were already mutated in the p53 signature and the adjacent FTE (Fig. 2f–g). Importantly, in two additional patients for whom DNA extraction from the paraffin-embedded FTE was possible, we showed that the mutations occurring in the fully established HGSOCS were also present in the FTE (Supplementary Fig. S5).

### 3.4 Expansion of FTE Cells Strongly Expressing SOX2 is a Feature of HGSOCs

The profound increase in the number of SOX2-expressing cells in the FTE of our index patient was in sharp contrast to the rare SOX2 expression in the FTE of patients with benign conditions. Paradoxically, SOX2 expression in tumor cells was almost absent (Supplementary Figs. S5 and S6). The increase in SOX2 expression in potential precursor cells is particularly intriguing since it is one of only four factors required for acquisition of stem cell properties by terminally differentiated cells (Takahashi and Yamanaka, 2006) with important implication for acquisition of pluripotency, the ability of a cell to differentiate into different cell types, and susceptibility to tumor formation.

**Fig. 3:**
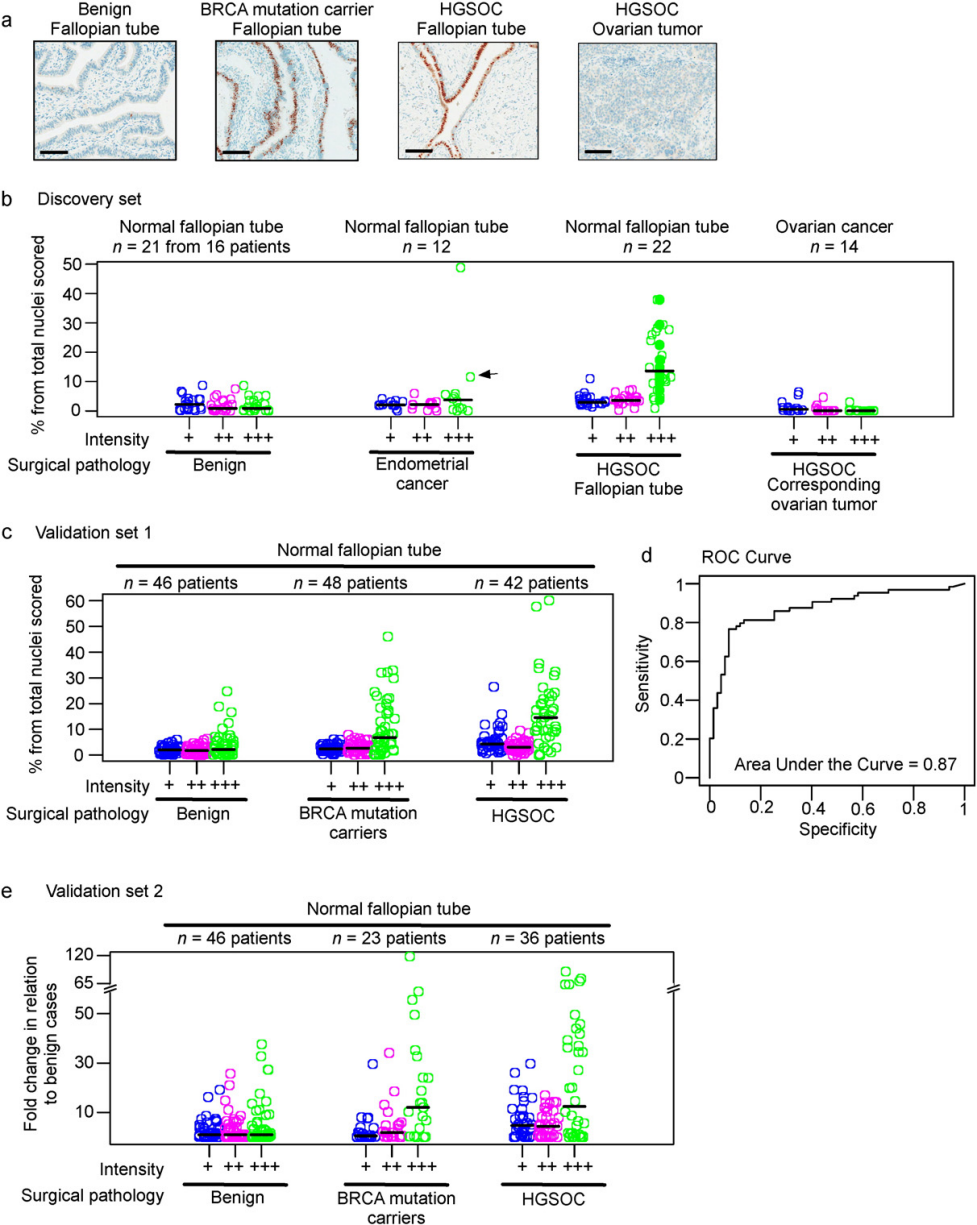
Overexpression of SOX2 is a biomarker for pre-neoplastic detection of HGSOC. a. Representative SOX2 immunohistochemistry images for the FTE of the indicated samples are presented. Scale bars = 100 μm. b. Percentages (y-axis), median percentage (horizontal black bars) and intensity (x-axis) of SOX2 staining in the normal FTE of women with benign conditions, endometrial cancer or HGSOCs and in the paired HGSOC tumors. Solid circles represent the FTE from cancers harboring rare variants and mutations in the BB5 region. The black arrow indicates a case of high-grade serous endometrial cancer. c. Power calculations were used to determine the required case number for the validation set based on the data from the discovery set (see Supplementary methods). Data from an independent set of 88 cases as well as an additional 48 BRCA1 or BRCA2 mutation carriers who underwent prophylactic excision of the fallopian tubes are presented. d. The receiver operating characteristics (ROC) curve is presented for the combined data presented in panels b and c. e. Data from a further independent set of cases from a second institute (CNIO) are presented. Data are presented as fold change relative to benign cases. A y-axis break was added to facilitate comparison with the data presented in b and c.

We next compared SOX2 expression in the nuclei of normal FTE of patients with benign conditions and patients with endometrial cancer (age-matched cases) and the normal FTE and corresponding ovarian tumors from patients diagnosed with HGSOC (Table 2, Supplementary Table 7). Automated image analysis of these samples demonstrated that the median SOX2 expression (intensity score of 3 +) in the normal appearing FTE from patients with HGSOCs was 17-fold higher than that in the normal FTE from patients with benign conditions (*p* < 2^− 16^, one-way analysis of variance [ANOVA] followed by Tukey's test). The median SOX2 expression significantly decreased in the corresponding, fully established ovarian tumors (*p* < 2^− 16^, one-way ANOVA followed by Tukey's test, Fig. 3a–b). We validated this observation on an independent set of fallopian tubes from 88 women with either HGSOCs (*n* = 42) or benign gynecological conditions (*n* = 46) and confirmed that a significant expansion of cells strongly expressing SOX2 was present in the benign FTE of women with HGSOCs (*p* < 2^− 16^, one-way ANOVA followed by Tukey's test, Fig. 3c). The magnitude of SOX2 overexpression was not significantly influenced by tumor stage (early versus late) or by whether or not the patient had chemotherapy prior to surgical excision (data not shown). In addition, fitting a binomial general linear model on the entire dataset revealed that strong SOX2 expression accurately predicted whether the fallopian tube was from an HGSOC patient or from a patient with benign pathology (*p* = 9.95^− 8^, logistic regression model). Fitting a receiver operating characteristic (ROC) curve to the data from the logistic regression analysis confirmed the high predictive power of strong SOX2 expression (Fig. 3d, area under the curve [AUC] = 0.87). This ubiquitous feature of the FTE of patients with HGSOCs has not been previously described. Furthermore, only eight out of 28 cases stained for p53 also showed a p53 signature indicating that SOX2 overexpression may occur prior to p53 dysregulation.

To test whether the expansion of SOX2-expressing cells occurred prior to HGSOC development, we analyzed the fallopian tubes of 48 women at high risk of developing ovarian cancer because they were BRCA1 or BRCA2 gene mutation carriers and therefore underwent prophylactic salpingo-oophorectomy (Fig. 3c, Table 2). This analysis showed that a significant expansion of cells strongly expressing SOX2 was present in the benign FTE of women who were BRCA1 or BRCA2 mutation carriers (*p* < 0.001, one-way ANOVA followed by Tukey's test).

Finally, to confirm the above findings, we repeated the analysis on an additional cohort of 98 patients from three independent institutes (Fig. 3e, Table 2 and Supplementary Table 7). This analysis further confirmed that a significant expansion of cells strongly expressing SOX2 occurred in the benign FTE of women who are BRCA1 or BRCA2 mutation carriers and in women who had HGSOCs (*p* = 0.018 and *p* < 0.001, respectively, one-way ANOVA followed by Tukey's test). Expression of SOX2 in the normal FTE of BRCA gene mutation carriers was independent of age and whether the mutation was present in the BRCA1 or BRCA2 (data not shown). History of prior breast cancer was available for 43 cases who were either BRCA1 or BRCA2 mutation carriers. Of those only 15 patients had history of prior breast cancer. There was no significant difference in SOX2 expression between cases that either had or did not have breast cancer.

### 3.5 The BB5 Region Is a Repressor of SOX2 Expression

Specific sequences of non-coding regions in the genome are capable of regulating the expression of neighboring genes. Several lines of evidence strongly suggested that the BB5 region acted as a repressor of SOX2 expression. First, the BB5 mutation occurred in a 12-nucleotide DNA sequence (Supplementary Fig. S7A) that matched the previously reported regulatory motif model UW.0169 that was discovered by genomic DNaseI footprinting (Neph et al., 2012). We used an in-vivo reporter assay to test whether this motif changed the expression of a fluorescent protein and, therefore, was potentially functional (Supplementary Fig. S7B–E). Such standard reporter assays are traditionally performed in cultured cells. In this study we conducted the assay in live chicken embryos to gain additional information about the time and location of the activity of the BB5 region in regulating fluorescent protein (the reporter) expression. This analysis confirmed that the motif consisting of the BB5 nucleotide acted as a repressor and that mutation of the BB5 nucleotide significantly relieved this repressor activity (Supplementary Fig. S7B–E). Importantly, the protein fluorescence partially co-localized with cells that strongly expressed endogenous Sox2 in the chicken embryo and, therefore, implied that the element was plausible for regulating Sox2 expression. Second, we performed CRISPR-Cas9-mediated genome deletion, a method to remove a number of nucleotides from a target sequence in the genome, and deleted multiple essential nucleotides included in the motif model UW.0169 (Supplementary Fig. S7F) (Ran et al., 2013) in a human cell model. This induced a significant reduction in H3K27 acetylation, an indicator of open chromatin, and a significant and sustained increase in SOX2 expression in human cells (Supplementary Fig. S7G, Supplementary Table 6). Thus, this unequivocally confirmed that the element acted as a repressor of SOX2 expression in a human cell model. Third, mining the ENCODE data for potential transcription factor binding at this element revealed several factors that are known to be involved in regulating the stem cell state, such as YY1, NANOG, OCT4 and EZH2 (Supplementary Fig. S7F). Since EZH2, the catalytic subunit of the Polycomb repressor complex 2, is known to be involved in repressing gene expression by inducing H3K27 trimethylation at regulatory elements, we performed ChIP-sequencing on primary cultured fallopian tube epithelial cells to test whether EZH2 occupied the BB5 element or other elements that were sites of somatic mutations in other HGSOCs. This analysis confirmed that EZH2 occupied the BB5 element and several others (Supplementary Fig. S8A, Supplementary Table 11). In addition, the enzymatic inhibition of EZH2 resulted in a significant reduction in H3K27 tri-methylation (Supplementary Fig. S8B) indicating that the localization of the EZH2 at the BB5 element was indeed functional. In summary, the above data confirmed that the BB5 region is a repressor of SOX2 expression and, when mutated, releases SOX2 repression.

**Fig. 4:**
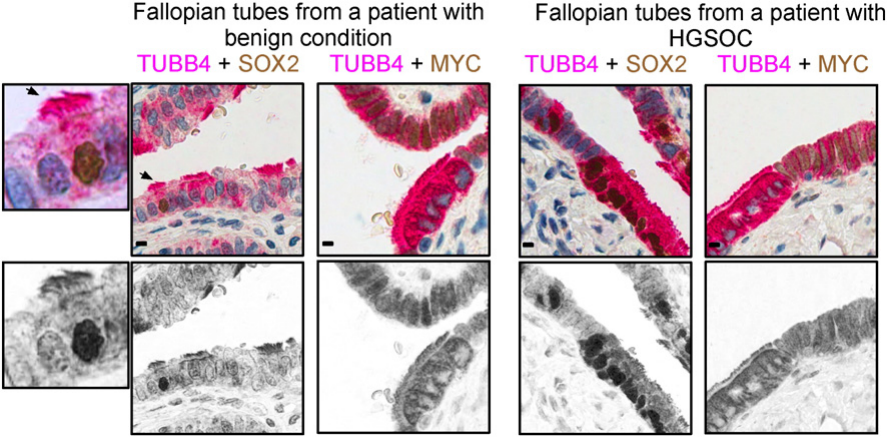
SOX2 represses MYC and PAX8 expression at the secretory-ciliated FTE cell interface. Representative immunohistochemistry images of FTE double stained with the indicated antibodies (*n* = 10). TUBB4 was used as a marker for ciliated cells. Scale bars = 10 μm. Arrows indicate cells that are presented in higher magnifications.

As previously mentioned, SOX2 is part of the four essential stem cells transcription factors (SOX2, OCT4, c-Myc, KLF4), also known as Yamanaka factors (Takahashi et al., 2007), which are highly expressed in embryonic stem cells and that are sufficient to induce pluripotency in human somatic cells. We, therefore, investigated the expression of the Yamanaka factors in the FTE and observed a mutually exclusive expression pattern of SOX2 and MYC. In the FTE of benign cases, MYC appeared to be exclusively and ubiquitously expressed in non-ciliated cells whilst SOX2 was only expressed at low levels in a minor population of ciliated cells (Fig. 4). In contrast, in the FTE from HGSOC cases MYC maintained a similar expression pattern in non-ciliated cells, whereas SOX2 was strongly expressed in the majority of ciliated cells (Fig. 4, Supplementary Fig. S9A–B). Furthermore, the analysis of 209 HGSOCs revealed that MYC over-expression was accompanied by the loss of SOX2 expression and vice versa (*p* < 0.001, Fisher Exact test, Supplementary Fig. S9C). Thus, SOX2 may repress MYC in differentiating FTE cells and in a fraction of SOX2-expressing tumors. Consistent with our proposed role of SOX2 in FTE cells, its ectopic expression significantly reduced expression of MYC and PAX8, an important transcription factor during embryogenesis of Müllerian tissues, in primary cultured FTE cells and SKOv3 ovarian cancer cells to undetectable levels by immunostaining (Supplementary Fig. S10). These findings are consistent with the known role of SOX2 in inducing pluripotency and may have important implications in understanding the genesis of epithelial ovarian cancers.

## 4 Discussion

Less than a third of ovarian cancer patients are diagnosed at an early, hence potentially curable, stage. The currently available detection options are patient driven symptom awareness and screening using combination of a serum marker (CA125) and imaging by ultrasound methods. Advances have been made to divert from the single threshold rule for CA125 to more complex algorithms in order to improve performance and facilitate early treatment. Jacobs et al. (2016) recently reported results of the UK Collaborative Trial of Ovarian Cancer Screening (UKCTOCS), a study in which over 200,000 postmenopausal women were enrolled to assess whether screening is effective for improving early ovarian cancer diagnosis and survival. Results indicated a limited mortality reduction in the primary analysis (Jacobs et al., 2016). This, again, underscores the need for effective biomarkers for early detection.

Recent work strongly suggests that the FTE is a common site of a large fraction of high-grade serous pelvic cancers (Perets et al., 2013). Inducing mutations in *TP53*, *BRCA1-BRCA2*, and *PTEN* in secretory cells in the FTE of mice resulted in the development of HGSOCs (Perets et al., 2013). However, there continues to be a need for novel molecular markers of premalignant transformation that could be used for early detection. Current screening methods only enable the detection of established invasive ovarian cancers. Mathematical modeling of tumor occurrence in BRCA1-BRCA2 mutations carriers suggests that tumors remain occult for up to four years (Brown and Palmer, 2009). This duration represents an unutilized opportunity for premalignant or early stage disease detection because of the limitations of existing markers. In addition, patients with a family history of ovarian cancer who don't have affected living relatives are not eligible for BRCA1-BRCA2 mutation testing. Furthermore, in many cases of familial ovarian malignancies BRCA testing is negative. Subsequently, these patients are ineligible for risk-reducing salpingo-oophorectomy.

Lack of specificity of TP53 overexpression (the so called p53 signature) in the fallopian tube (Lee et al., 2007) undermines its potential use in early detection. In contrast, we have shown that SOX2 overexpression has high specificity and sensitivity as evidenced by the high AUC in our ROC analysis. In addition, unlike the p53 signature which only involves a limited number of cells, SOX2 overexpression occupies a large surface area of the fallopian tube. This makes SOX2 overexpression amenable for detection. Safe screening methods that exploit such feature would need to be developed in both BRCA mutation carriers and patients with HGSOC. The use of cell-penetrating peptides or antibodies that specifically recognize SOX2 could be labeled with PET imaging tracers to enable early, non-invasive detection in patients. In addition, fallopian tube catheterization for obtaining cytobrush specimens for quantifying SOX2 expression is also possible. Such methods could be tested in clinical trials for early detection in young women with BRCA1-BRCA2 mutations who do not wish to undergo prophylactic excision of both tubes and ovaries. Finally, as SOX2 is a potent transcription factor, it is highly likely that its aberrant expression is associated with co-expression of secreted proteins. The future discovery of such proteins could enable biomarker screening using blood sampling.

In summary, in this study we demonstrated that SOX2 overexpression occurs in a fraction of women with BRCA1 and BRCA2 mutations prior to ovarian cancer initiation and in the majority of patients with HGSOCs irrespective of tumor stage. These findings could be exploited for filling the current gap in early detection strategies for ovarian cancer. We believe that this is the first report of the expansion of SOX2-expressing cells in the FTE of HGSOCs. This finding has important implications, as it provides a potentially powerful tool for screening for HGSOCs. Utilizing our findings as potential biomarker should take high priority.

The following are the supplementary data related to this article.

A file containing supplementary figures. Image 1

Supplementary Table 1 Description of genomes analyzed for patient 11152. Supplementary Table 1

Supplementary Table 2 The coordinates and functional annotations of the 750 mutations are presented. Supplementary Table 2

Supplementary Table 3 The complete analysis results for enrichment in ontologies of biological processes using the list of progeny variants as a background comparator. Supplementary Table 3

Supplementary Table 4 The gene symbols that correspond to the identified five significantly enriched ontologies are presented along with the distance in bases from the mapped mutation to the gene's transcription start site. Supplementary Table 4

Supplementary Table 5 The clinical details of patients who donated samples used for targeted sequencing. Supplementary Table 5

Supplementary Table 6 The 861 rare variants identified by targeted sequencing of samples from 33 patients are presented. Supplementary Table 6

Supplementary Table 7 The coordinates of the somatic mutations identified at the 40 kb region flanking *SOX2* in HGSOCs. Supplementary Table 7

Supplementary Table 8 The clinical details of patients who donated the samples used for SOX2 immunohistochemistry are presented. Supplementary Table 8

Supplementary Table 9 List of transfections and summary results for CRISPR experiments. Supplementary Table 9

Supplementary Table 10 Coordinates of the areas of focal enrichment (peaks) for the EZH2 ChIP-sequencing analysis in the 1.6 Mb region around the SOX2 transcription start site. Supplementary Table 10

Supplementary Table 11 Primers used for sequencing individual variants in patients with HGSOCs. Supplementary Table 11

Supplementary video A video showing the laparoscopy finding for the sites that were sampled at presentation for whole genome sequencing prior to chemotherapy and the findings following the administration of three cycles of chemotherapy. Note the complete macroscopic resolution of the tumors. Supplementary video

## Funding Sources

This work is funded by the Medical Research Council (H8RSRS00), Ovarian Cancer Action (HER00070), the Oxford Biomedical Research Centre, the National Institute for Health Research (HJRWAC05) and the Experimental Cancer Medicine Centre. MJG is recipient of a research contract from the Instituto de Salud Carlos III of the Ministerio Español de Sanidad y Consumo (Miguel Servet tipo II Program, CPII 13-00047). C.Y. acknowledges the support of an MRC New Investigator Research Grant (Ref No. MR-L001411-1) and the Wellcome Trust Core Award Grant Number 090532-Z-09-Z.

## Conflicts of Interests

R.T., B.A.P., R.D., D.M.H., R.Y.Z., R,K., and K.D. own stock in Complete Genomics, Inc. All other authors have no conflict of interest to declare.

## Contributions

A.A.A. conceived the project. All other authors designed and conducted experiments. A.A.A., K.H., F.M., S.H.G., and C.Y. wrote the manuscript. All authors contributed to the final manuscript.

## Figures and Tables

**Table 1:** Patient baseline data for targeted sequencing analysis. The clinical details of patients who donated samples used for targeted sequencing are presented in this table.

Patients for targeted sequencing	49
Age at surgery	
Mean (median)	65.3 (66)
Min–max	40–82
< 60 years	20.4%
> 60 years	79.6%
Site of primary disease	
Ovary	29 (59%)
Tube	10 (20%)
Peritoneum	8 (16%)
Other^a^	2 (4%)
Tumor status	
Primary	19 (39%)
IDS	15 (31%)
Post-NACT	12 (24%)
Recurrence	3 (6%)
Biopsy site	
Ovary	17
Peritoneum^b^	15
Omentum	17
Other^c^	4
Normal tissue or blood^d^	24
Tumor stage^e^	
I–II	10 (20%)
III–IV	39 (80%)

Data are *n* (%). Disease stages I and II indicate early disease, stages III and IV indicate advanced disease. NACT = neoadjuvant chemotherapy. IDS = interval debulking surgery (e.g. after three or four cycles of NACT).

a Ambivalent histology includes one case of tubal or ovarian origin and a case of ovarian or endometrial origin.

b Peritoneum includes diaphragmatic peritoneum and small bowel serosa and mesentery.

c Other biopsy sites include spleen, parasacral tumor and falciform ligament.

d Normal tissue includes primary cell lines generated from normal fallopian tube epithelium.

e For staging we used the FIGO classification valid at time of initial diagnosis.

**Table 2:** Patient characteristics for immunohistochemistry studies. The clinical details of patients who donated the samples used for SOX2 immunohistochemistry are presented.

Patients for IHC staining *n* = 279	Benign	HGSOC	BRCA mutation carrier
Patient cohort	108	100	71
Discovery cohort	16	22^a^	
Validation cohort	92	78	71
Age at surgery			
Mean (median)	48.0 (48)	62.7 (63)	50.7 (48)
Min–max	28–81	32–94	29–81
Site of primary disease			
Ovary		79 (79%)	
Tube		10 (10%)	
Peritoneum		5 (5%)	
Other^b^		6 (6%)	
Tumor status			
Primary		60 (60%)	
IDS		15 (15%)	
Post-NACT		19 (19)	
Tumor stage^c^			
I–II		26 (26%)	
III–IV		69 (69%)	

Data are *n* (%) or mean (median), unless otherwise indicated. Disease stages I and II indicate early disease, stages III and IV indicate advanced disease. IDS = Interval debulking surgery (e.g. after three or four cycles of NACT). NACT = neoadjuvant chemotherapy.

a These patients were also included in targeted sequencing study.

b Ambivalent histology cohort includes four cases of ovarian or tubal origin and two cases of ovarian or peritoneal origin.

c For staging we used the FIGO classification valid at time of initial diagnosis.
