# Supplementary material for: Premalignant SOX2 overexpression in the fallopian tubes of ovarian cancer patients: Discovery and validation studies
Source: eBioMedicine. 2016 Jul 2;10:137–49. doi: 10.1016/j.ebiom.2016.06.048 (PMC5006641; doi:10.1016/j.ebiom.2016.06.048)
Supplement: Supplementary file 1 — A file containing supplementary figures. [file mmc1.docx]

**Premalignant SOX2 overexpression in the fallopian tubes of ovarian cancer patients: discovery and validation studies**

SUPPLEMENTARY APPENDIX

Karin Hellner, MD^†1,2^, Fabrizio Miranda, PhD^†1,2^, Donatien Chedom Fotso, PhD^†1,2,15^, Sandra Herrero-Gonzalez, PhD^†1,2^, Daniel M. Hayden, PhD ^†3^, Rick Tearle, PhD ^†3^, Mara Artibani, PhD^1,2,4^, Mohammad KaramiNejadRanjbar^1,2^, Ruth Williams, PhD ^4^, Kezia Gaitskell, MD^1,2^, Samar Elorbany, MD^1,2^, Ruoyan Xu, PhD^1,2^, Alex Laios, MD^1,2^, Petronela Buiga, MD^1,2^, Karim Ahmed^5^, Sunanda Dhar, MD^6^, Rebecca Yu Zhang. PhD^3^, Leticia Campo, PhD^7^, Kevin A. Myers, PhD^7^, María Lozano, PhD^8^, María Ruiz-Miró, PhD^9^, Sónia Gatius, PhD^10^, Alba Mota, PhD^11,12^, Gema Moreno-Bueno, PhD^11,12^, Xavier Matias-Guiu, PhD^10^, Javier Benítez, PhD^13,14^, Lorna Witty, PhD^15^, Prof Gil McVean, PhD^15^, Prof Simon Leedham, MD^15^, Prof Ian Tomlinson, MD^15^, Radoje Drmanac, PhD^3,16^, Prof Jean-Baptiste Cazier, PhD^7,17^, Robert Klein, PhD^3^, Kevin Dunne, PhD^3^, Prof Robert C. Bast Jr, MD^18^, Prof Stephen H. Kennedy MD^2^, Prof Bassim Hassan, MD^19^, Stefano Lise, PhD^15^, María José Garcia, PhD^13,14^, Brock A. Peters, PhD^3,16^, Christopher Yau, PhD^15,20^, Prof Tatjana Sauka-Spengler, PhD^4^, Prof Ahmed Ashour Ahmed, MD*^1,2^

^1^Ovarian Cancer Cell Laboratory, Weatherall Institute of Molecular Medicine, University of Oxford, Headington, Oxford, OX3 9DS, UK.

^2^Nuffield Department of Obstetrics and Gynaecology, University of Oxford, Women’s Centre, John Radcliffe Hospital, Oxford, OX3 9DU, UK.

^3^Complete Genomics, Inc., 2071 Stierlin Ct., Mountain View, CA 94043.

^4^University of Oxford, Gene Regulatory Networks in Development and Disease Laboratory, Weatherall Institute of Molecular Medicine, Radcliffe Department of Medicine, Oxford, OX3 9DS, UK.

^5^Trinity College, University of Cambridge, Cambridge, UK, CB2 1TQ.

^6^Department of Histopathology, Oxford University Hospitals, Oxford, OX3 9DU, UK.

^7^Department of Oncology, University of Oxford, Old Road Campus Research Building, Roosevelt Drive, Oxford, OX3 7DQ, UK.

^8^Histopathology Core Unit, Spanish National Cancer Research Centre (CNIO), Madrid, Spain

^9^Biobank, Institut de Recerca Biomèdica Lleida (IRBLLEIDA), Lleida, Spain.

^10^Department of Pathology and Molecular Genetics, Hospital Universitari Arnau de Vilanova, University of Lleida, IRBLLEIDA, Lleida, Spain.

^11^Departament of Biochemistry, Universidad Autonoma de Madrid (UAM), Instituto de Investigaciones Biomédicas “Alberto Sols” (CSIC-UAM), IdiPAZ, Madrid, Spain

^12^MD Anderson international Foundation, Madrid, Spain

^13^Human Genetics Group, Spanish National Cancer Research Center (CNIO), Madrid, Spain

^14^Biomedical Network Research Centre on Rare Diseases (CIBERER)

^15^Wellcome Trust Centre for Human Genetics, and NIHR Biomedical Research Centre, Roosevelt Drive, Oxford, OX3 7BN, UK.

^16^BGI-Shenzhen, Shenzhen, China.

^17^Centre for Computational Biology, University of Birmingham, B15 2TT, Edgbaston.

^18^Department of Experimental Therapeutics, M.D. Anderson Cancer Center, University of Texas, USA.

^19^Tumour Growth Control Group, Sir William Dunn School of Pathology, South Parks Road, Oxford, OX1 3RE, UK.

^20^Department of Statistics, 1 South Parks Road, Oxford, OX1 3TG, UK.

† These authors contributed equally to this work

* Correspondence to ahmed.ahmed@obs-gyn.ox.ac.uk

**Contents**

| Supplementary figures and figure legends | Page 2 |
| --- | --- |
| Description of supplementary tables and video | Page 12 |
|  |  |

**Supplementary Figures**

**Fig. S1: Strategy for laser-capture microdissection and validation**

A total of 84 tumor islets and 41 stroma islets were microdissected by using individual caps for each islet. For each microdissected islet, at least five images were obtained for documentation. Islets were subjected to LFR WGS. **A.** Shown are representative images of one microdissected islet at low magnification, high magnification, after microdissection, and in the collection cap. Scale bars = 150 μm. **B.** A diagram depicting the workflow and the number of genomes used at each stage of the study.


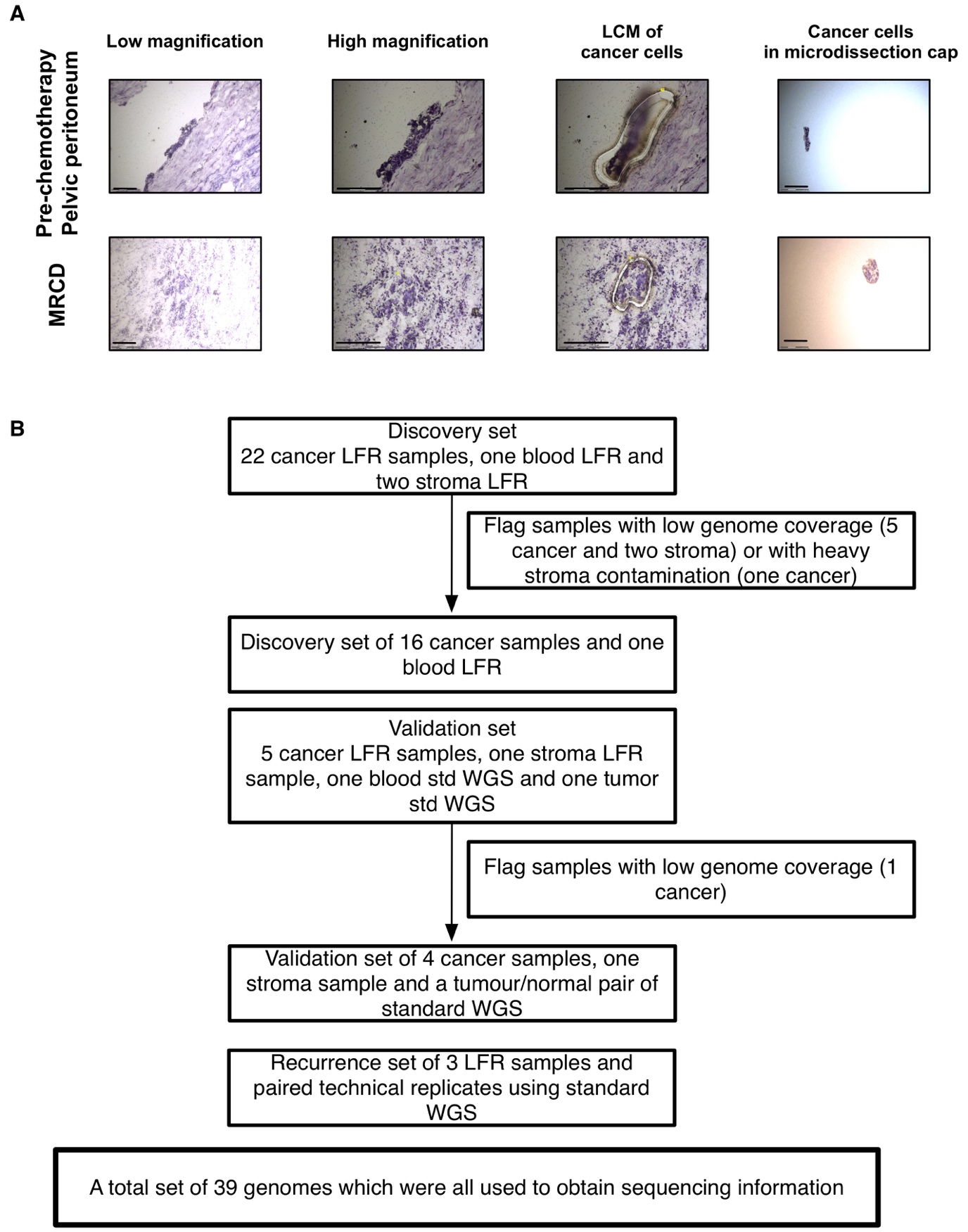


**Fig. S2: Noncoding core mutations cluster at potential enhancers of stem cell differentiation genes.**

**A.** A diagram depicting the validation strategy of the identified variants. Further validation using 1) WGS of unamplified lymphocyte DNA, 2) LFR sequencing of a further stroma sample (85 % genome coverage) and 3) targeted sequencing (at 50X read depth) of all variants in germline DNA excluded the possibility of these being germline variants. Repeat deep targeted sequencing (450X) of all variants on germline DNA identified two that were compatible with mosaic state (chr21: 9,833,828_C/A; 204 out of 2542 reads and chr7: 61,874,043_C/A; 273 out of 1115 reads). Other variants (*n* = 7) were either present at a very low frequency (< 1: 100) or were not confirmed by subsequent standard sequencing. Furthermore, extensive validation using 1) LFR WGS of three additional tumor islets, 2) standard WGS of bulk omental tumor and 3) deep targeted sequencing of all variants on DNA from two bulk tumor samples (positions B and C) confirmed the presence of all 750 mutations. **B.** The fold enrichment and the *log* number of variants that supported any significantly enriched ontologies are presented. The top five ontologies with greater than 2-fold enrichment over the background are also indicated.

**
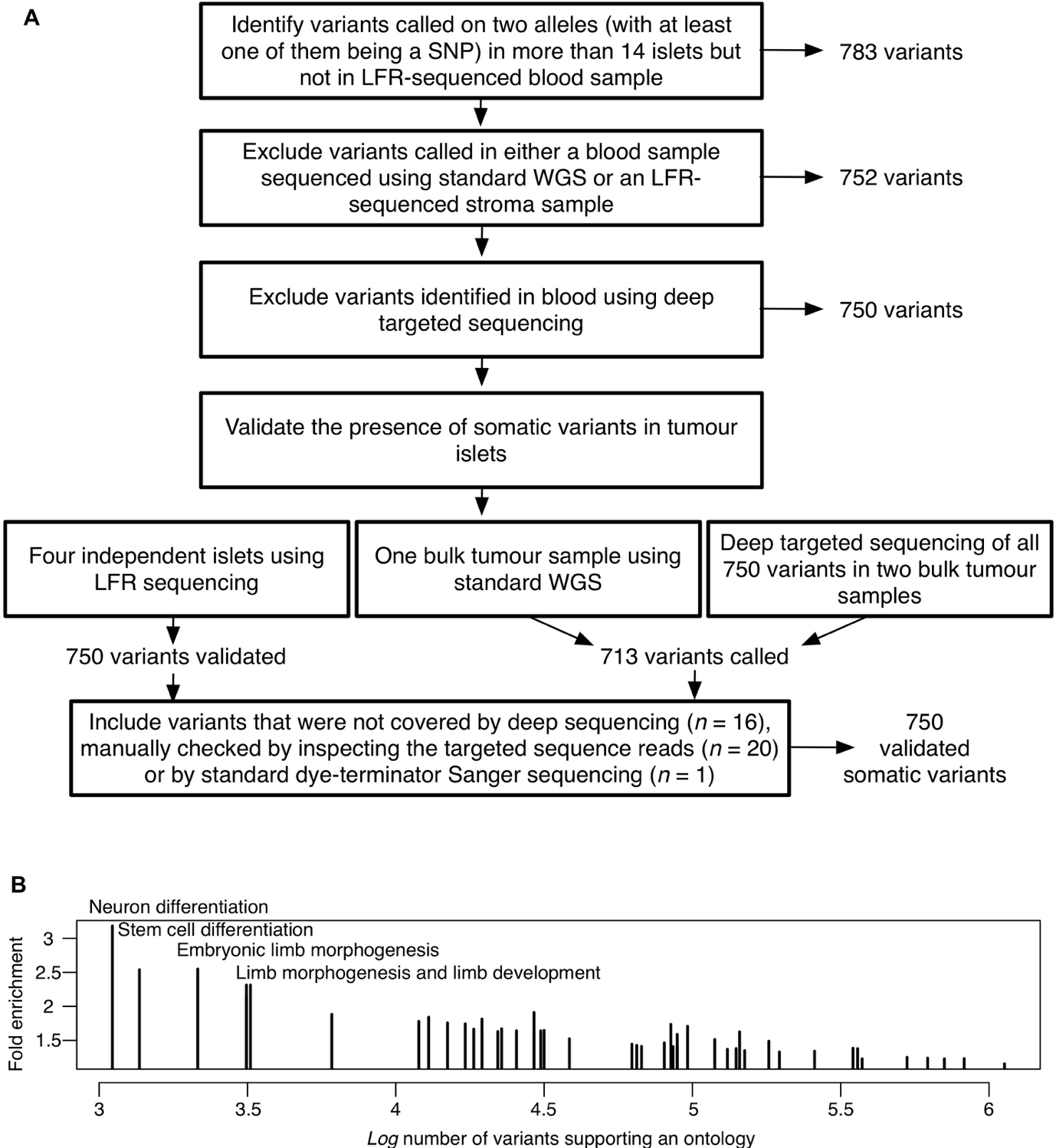
**

**Fig. S3: Analysis strategy for identifying enriched gene ontologies**

A flowchart illustrating the steps of our validation approach of the stem cell differentiation ontology enrichment The 750 variants shared by more than 90 % of tumor islets, hence called ancestor variants, were mapped to the potential gene regulatory regions. Initially, to test for enrichment, somatic variants that were called in any two tumor islets (*n =* 330,811, progeny variants used as a “background” comparator) were used as a background comparator (BG). See supplementary methods for analysis details.

**
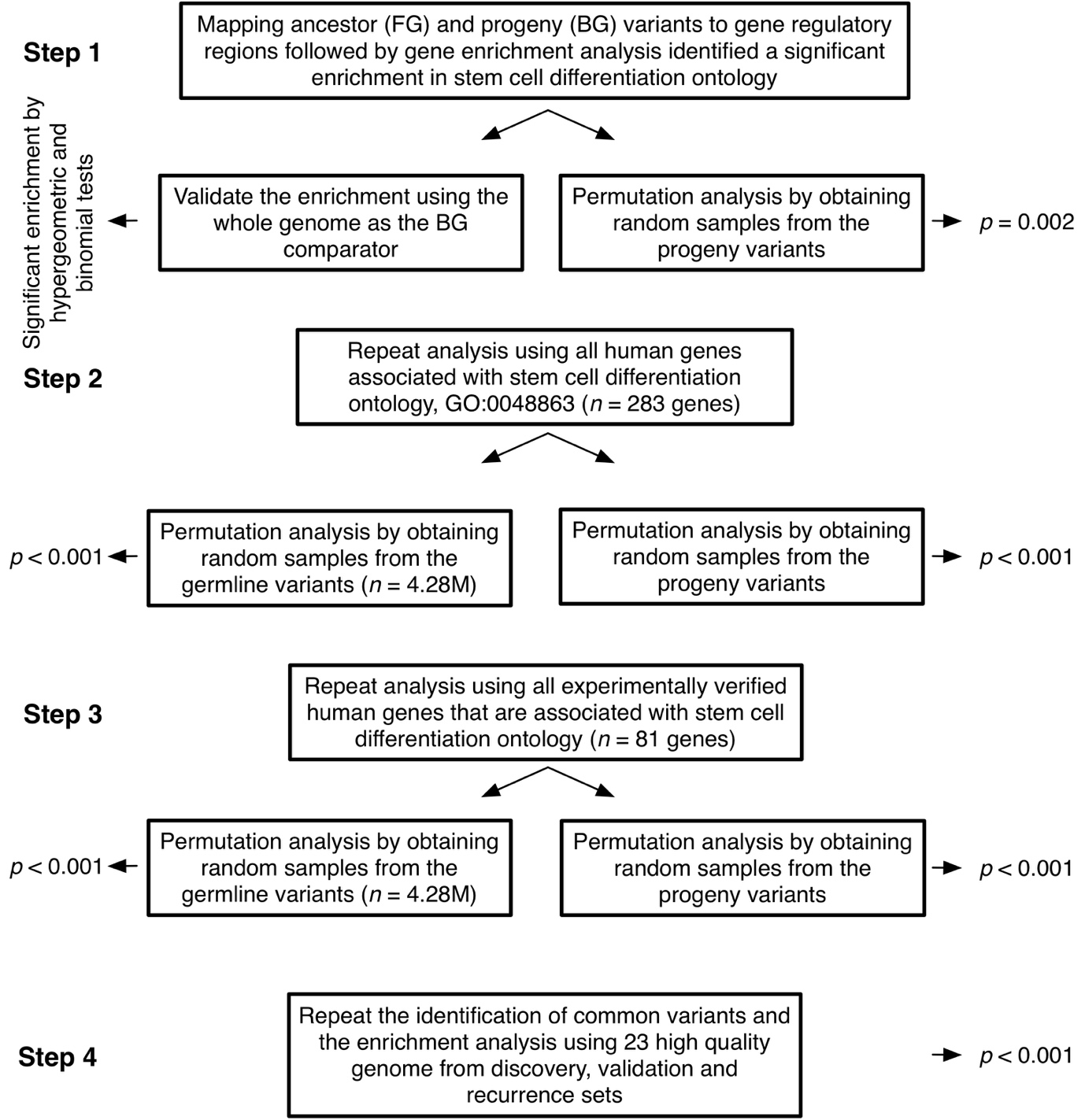
**

**Fig. S4: Rare variants cluster at biochemically active sites within a 2Mb region encompassing *SOX2***

Deep-targeted sequencing was performed on 33 HGSOCs, and 861 rare variants (not previously reported in the 1,000 Genomes Project) were identified. We tested the assumption that the region was enriched in variants that occupied functionally important elements by mapping the variants to biochemically active sites, as reported from the ENCODE project. The BB5 region was also significantly enriched (*p <* 0·01) in variants that overlapped with genomic sites previously reported to harbor biochemical activity (DNaseI hypersensitivity or transcription factor binding activity). Shown is the statistical significance (*log*10 1/*p*-value) of the enrichment of rare variants that fall in known biochemically active sites (orange bars) in 40 Kb moving windows. The locations of the BB nucleotides are shown, and the BB5 region is indicated by a horizontal magenta bar.

**
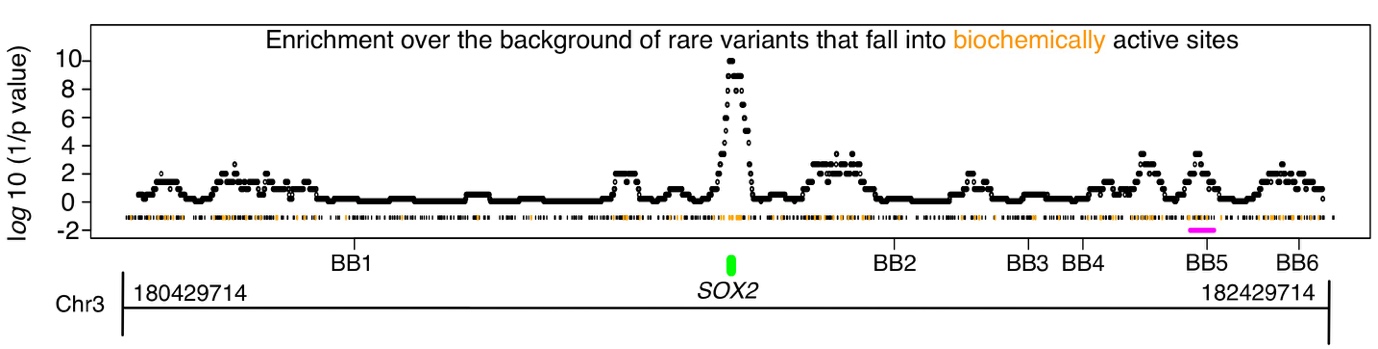
**

**Fig. S5: Mutations in the BB5 enhancer region in HGSOCs are associated with high SOX2 expression in the FTE**

**A-F.** The identified somatic mutations (A, B, D, E, F) or LOH (C) in the 40 Kb region containing BB5 in six HGSOCs (left panel) are shown. SOX2 immunohistochemistry (IHC) staining in the corresponding apparently normal FTE and (for C and E) the lack of SOX2 nuclear expression in the adjacent ovarian tumors (right panel) are also shown. Note that for patient 11249, macrodissection of the fallopian tube was possible. DNA sequencing from the FTE revealed that a heterozygous mutation found in the tumor was also present in the FTE and appeared to be homozygous.

**
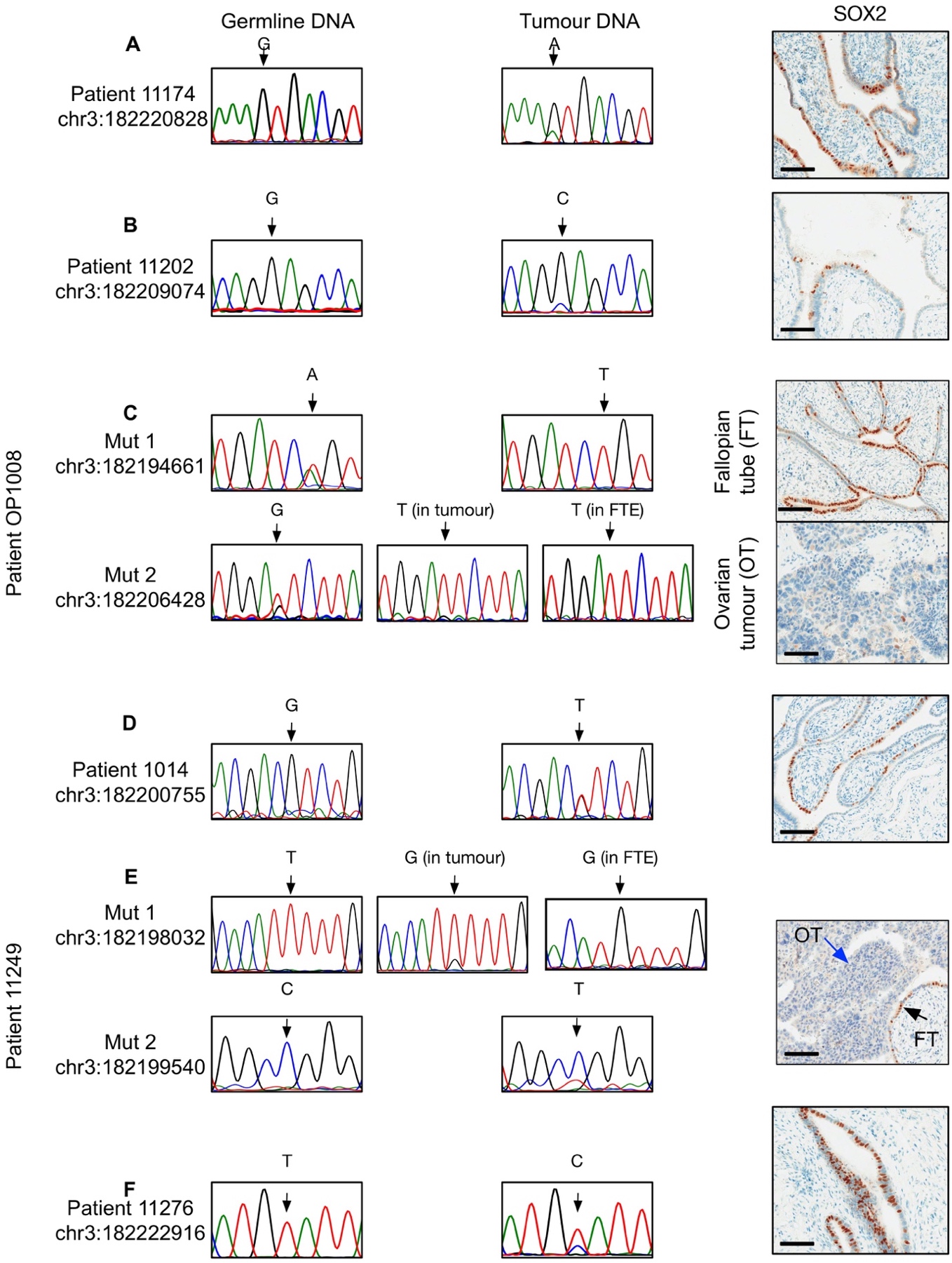
**

**Fig. S6: SOX2 expression in the FTE and tumor tissue samples in patient 11152**

**A.** Bar plot of the percentage of nuclei exhibiting strong (green), medium (magenta) or low SOX2 expression (blue) in the apparently normal FTE, tumor tissue prior to chemotherapy (Pre) in two omentum samples (OM1 and OM2) and one diaphragmatic peritoneum sample (DR), and following chemotherapy (Post) in the left ovarian tumor (LOT) of our index case patient 11152. **B**. IHC staining for SOX2, p53 and WT1 (an epithelial cell marker) in the FTE at the p53 signature, a pre-chemotherapy tumor islet (OM1) and a post-chemotherapy tumor islet (LOT) of the index case patient. Positive p53 and WT1 staining confirms that the SOX2-negative cells are cancer cells. Scale bars = 100 μm.

**
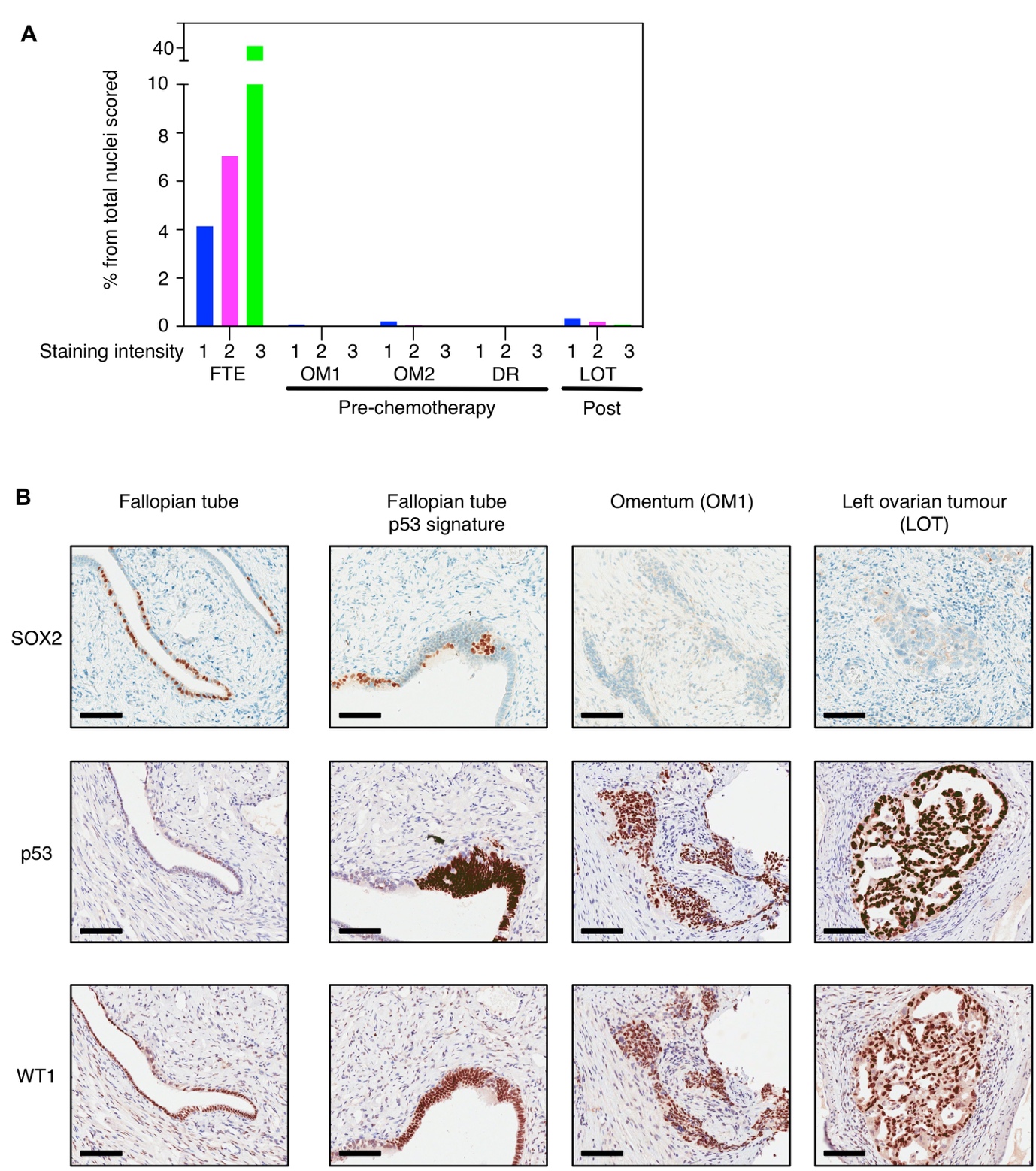
**

**Fig. S7: The BB5 nucleotide is located within a Sox2 regulatory element and represses SOX2 expression *in-vivo* in a chicken embryo model and mammalian cells**

**A.** Logo for sequence motif UW.0169. The letter height corresponds to the probability of occurrence of a nucleotide. At the BB5 nucleotide, strong preference (14-fold higher frequency) for the wild type C nucleotide over the mutant G nucleotide (observed frequencies C = 0·86 and G = 0·06) was identified. **B.** Activity of the BB5 wild type and mutant enhancer region in chicken embryos at somite stage nine (*n* = 8). **C.** The transfection of wild type and mutant plasmids on opposite sides of the same embryo, to reveal differences in activity, is shown. A typical image of ten repeats is presented. Note that the wild type enhancer controlled citrine expression predominantly within the neural tube, the endogenous site of Sox2 expression, in both the head and trunk regions, and in the delaminating neural crest cells. By contrast, the activity was dramatically decreased in the single-point (C/G) mutant version in the neural crest but was retained in the cell population within the head region at early stages as shown. Thus, this nucleotide possesses an important functional role in the regulatory activity of the element, and its function as repressor or enhancer is tissue specific. **D**. A systematic analysis of deletion mutants of the 1 Kb fragment encompassing the BB5 nucleotide. A diagram representing the genomic regions tested in the chicken embryo reporter assays. The magnitude of activity was estimated as weak (+), moderate (++) or strong (+++). **E.** The transfection of wild type and mutant plasmids containing the “test 3” region on opposite sides of the same embryo to indicate recovery of the activity after inducing the BB5 mutation. A representative image of five embryos is presented. Note that systematic truncations mutants of the 1 Kb fragment revealed that a 363 bp segment downstream of the BB5 nucleotide retained full enhancer activity. However, extending this segment to involve the BB5 nucleotide resulted in a significant loss of enhancer activity. Moreover, mutating the BB5 nucleotide from C to G in the extended segment to mimic the patient’s mutation significantly increased the enhancer activity, indicating that the BB5 element had a repressive function. **F.** CRISPR-mediated deletion of six nucleotides (magenta) downstream of the BB5 nucleotide. The protospacer adjacent motif (red) and representation of ENCODE data for transcription factor binding at this site is also shown. **G.** DNA electrophoresis and western blots for wild type HEK293 cells and mutant clone SH26A. Note the loss of overexpression that coincided with overgrowth of the wild-type subclone as indicated by the DNA electrophoresis (*n* of replicates indicated in supplementary table 9).


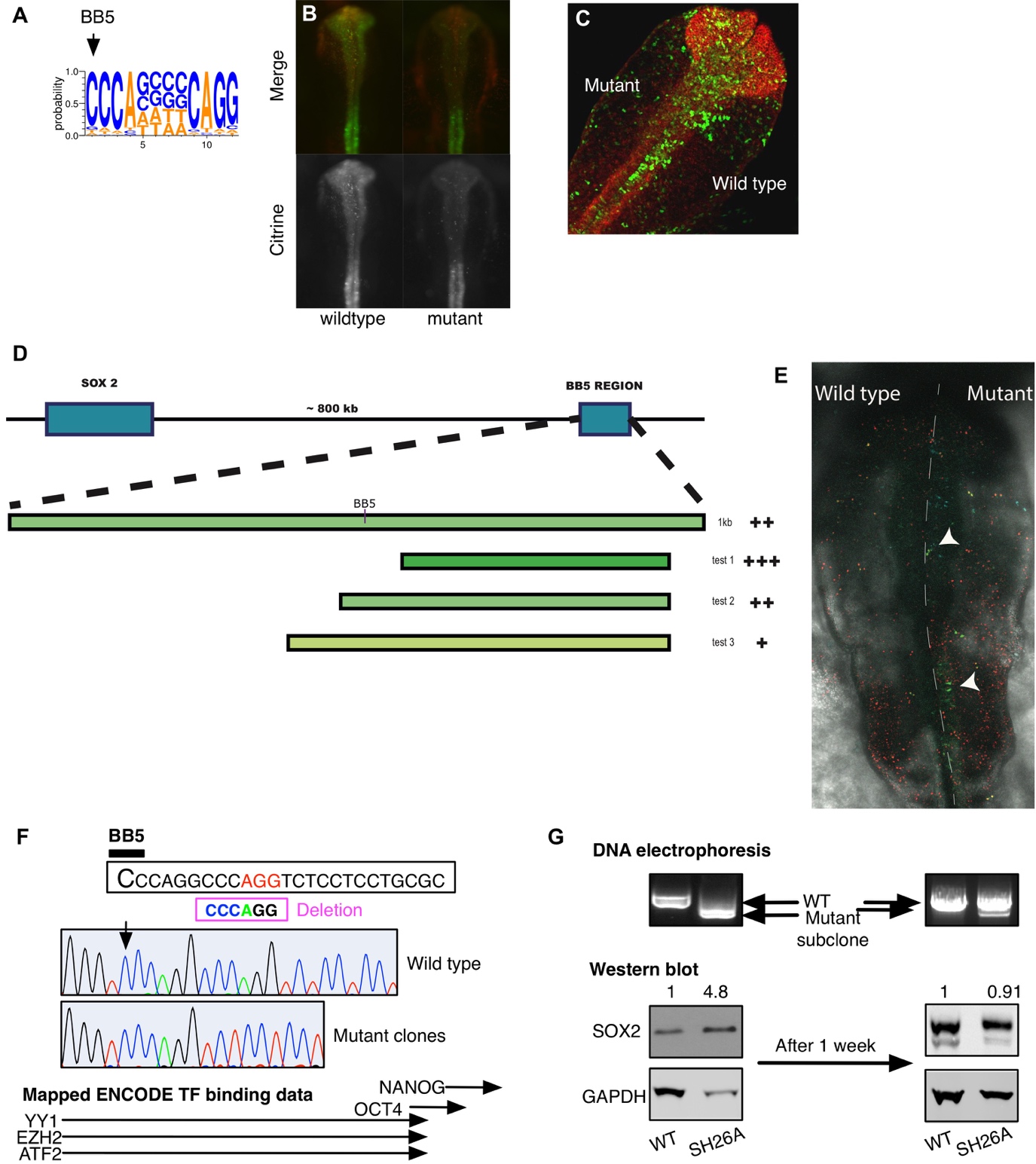


**Fig. S8: EZH2 occupies the BB5 repressor element**

**A.** Whole-genome ChIP-seq was performed on FTE cells using EZH2 antibody to detect areas of occupancy. The peaks presented are significantly enriched (*p* < 0·01) relative to those of input control. The location of the 11 identified somatic mutations found in our HGSOC cohort are also presented. **B.** ChIP-qPCR results indicating the significant reduction of H3K27me3 at the BB5 element following chemical inhibition of EZH2 (*n* = 3 patients).

**
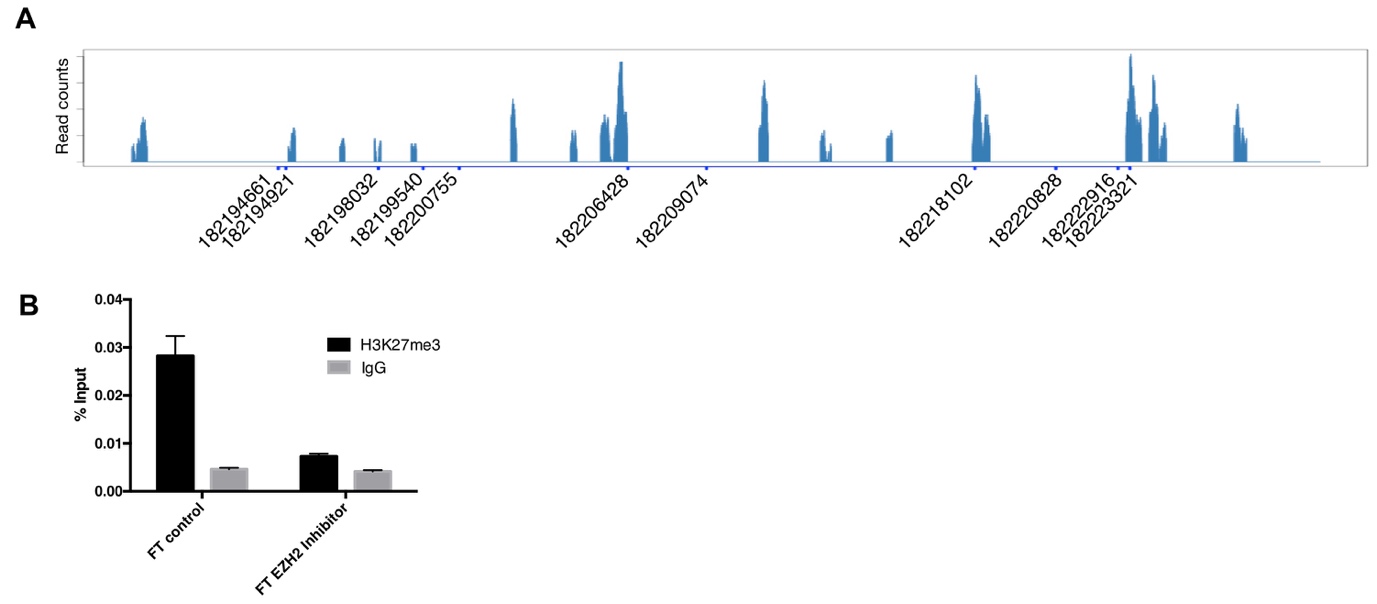
**

**Fig. S9: The expression of SOX2 and MYC in the FTE is mutually exclusive.**

**A-B.** Representative IHC images of the FTE stained for PAX8 (A), MYC or SOX2 (B) are shown. Arrows point to ciliated cells. Note that while PAX8 and MYC expression are limited to non-ciliated cells, SOX2 expression is exclusively in ciliated cells. **C.** Representative images of HGSOCs stained for SOX2 and MYC (*n* = 209). A summary table for the staining intensity is also presented.


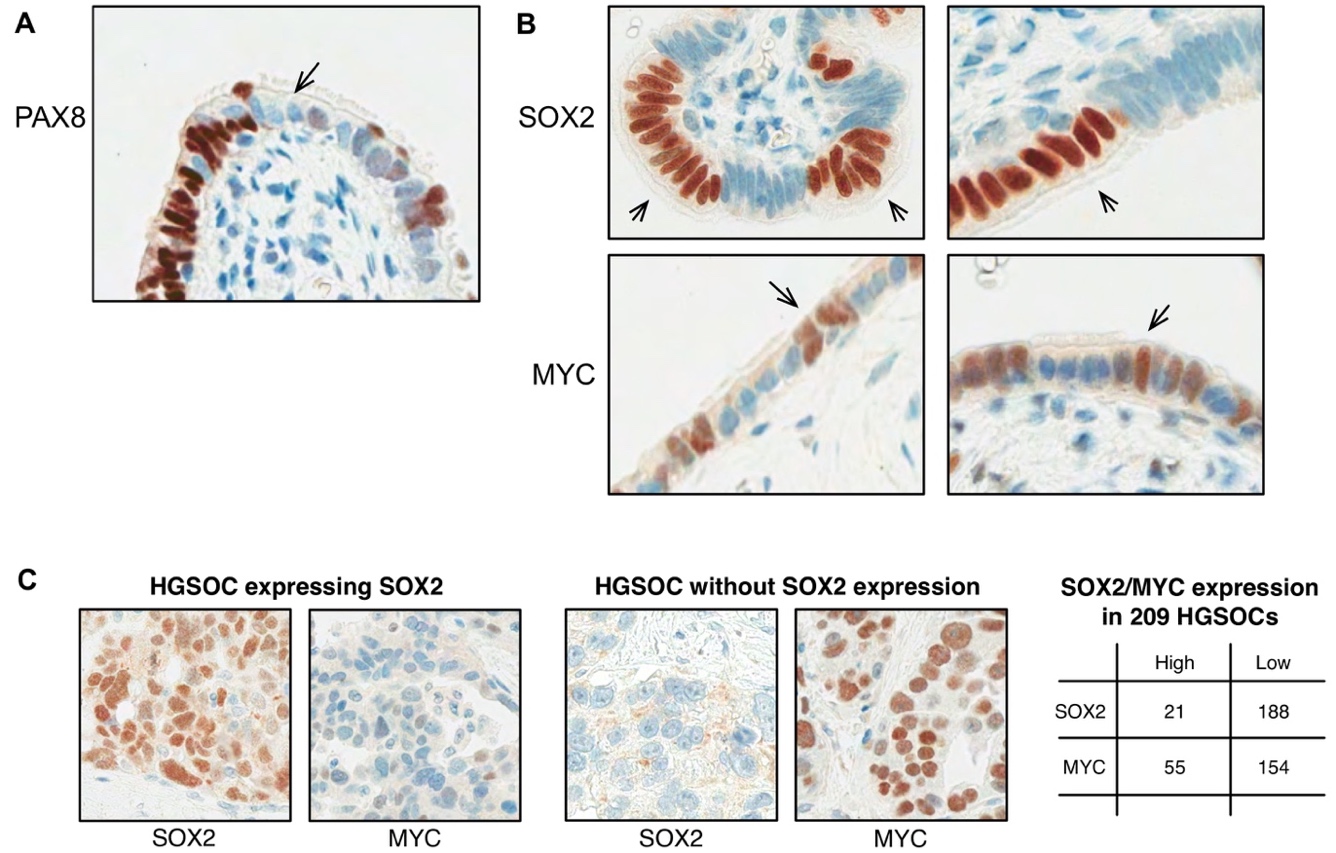


**Fig. S10: Ectopic expression of SOX2 represses MYC and PAX8 in primary cultured FTE cells and SKOv3 cells**

**A.** Primary cultured FTE cells were transduced with SOX2-expressing viruses for five days then fixed and stained for SOX2, PAX8 or MYC. Images were collected from 20 fields and the percentage of cells was obtained as indicated. Shown is the mean + s.e.m of percentages obtained from 290 cells. **B-C.** Immunofluorescence images of SKOv3 cells that were transduced with either SOX2-expressing lentiviruses or empty vector controls then fixed and stained using the indicated antibodies to test the change in expression of MYC (B) or PAX8 (C). Also shown are representative images of SOX2-overexpressing cells that were treated with SOX2-targeting siRNAs to deplete the ectopically expressed SOX2. Note the reappearance of PAX8 and MYC following SOX2 depletion.

**
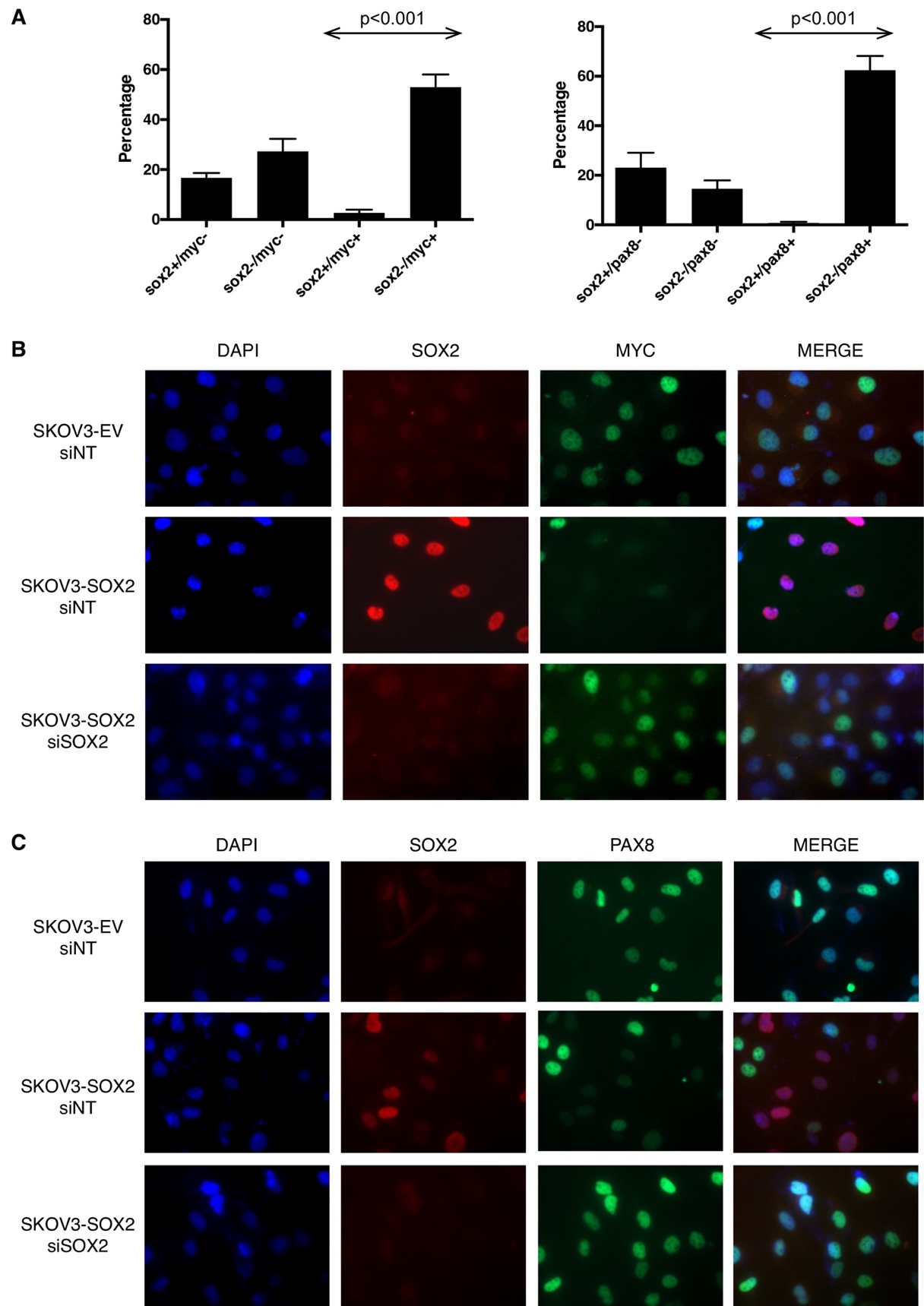
**

**Description of supplementary tables and videos**

This manuscript has 11 supplementary tables and a supplementary video as follows:

**Supplementary Table 1:**

Description of genomes analyzed for patient 11152.

**Supplementary Table 2:**

The coordinates and functional annotations of the 750 mutations are presented.

**Supplementary Table 3:**

The complete analysis results for enrichment in ontologies of biological processes using the list of progeny variants as a background comparator.

**Supplementary Table 4:**

The gene symbols that correspond to the identified five significantly enriched ontologies are presented along with the distance in bases from the mapped mutation to the gene’s transcription start site.

**Supplementary Table 5:**

The clinical details of patients who donated samples used for targeted sequencing

**Supplementary Table 6:**

The 861 rare variants identified by targeted sequencing of samples from 33 patients are presented.

**Supplementary Table 7:**

The coordinates of the somatic mutations identified at the 40 Kb region flanking *SOX2* in HGSOCs.

**Supplementary Table 8:**

The clinical details of patients who donated the samples used for SOX2 immunohistochemistry are presented.

**Supplementary Table 9:**

List of transfections and summary results for CRISPR experiments.

**Supplementary Table 10:**

Coordinates of the areas of focal enrichment (peaks) for the EZH2 ChIP-sequencing analysis in the 1.6 Mb region around the SOX2 transcription start site.

**Supplementary Table 11:**

Primers used for sequencing individual variants in patients with HGSOCs.

**Supplementary Video:**

A video showing the laparoscopy finding for the sites that were sampled at presentation for whole genome sequencing prior to chemotherapy and the findings following the administration of three cycles of chemotherapy. Note the complete macroscopic resolution of the tumors.
