# Supplementary figures and images for: Premalignant SOX2 overexpression in the fallopian tubes of ovarian cancer patients: Discovery and validation studies
Source: eBioMedicine. 2016 Jul 2;10:137–49. doi: 10.1016/j.ebiom.2016.06.048 (PMC5006641; doi:10.1016/j.ebiom.2016.06.048)

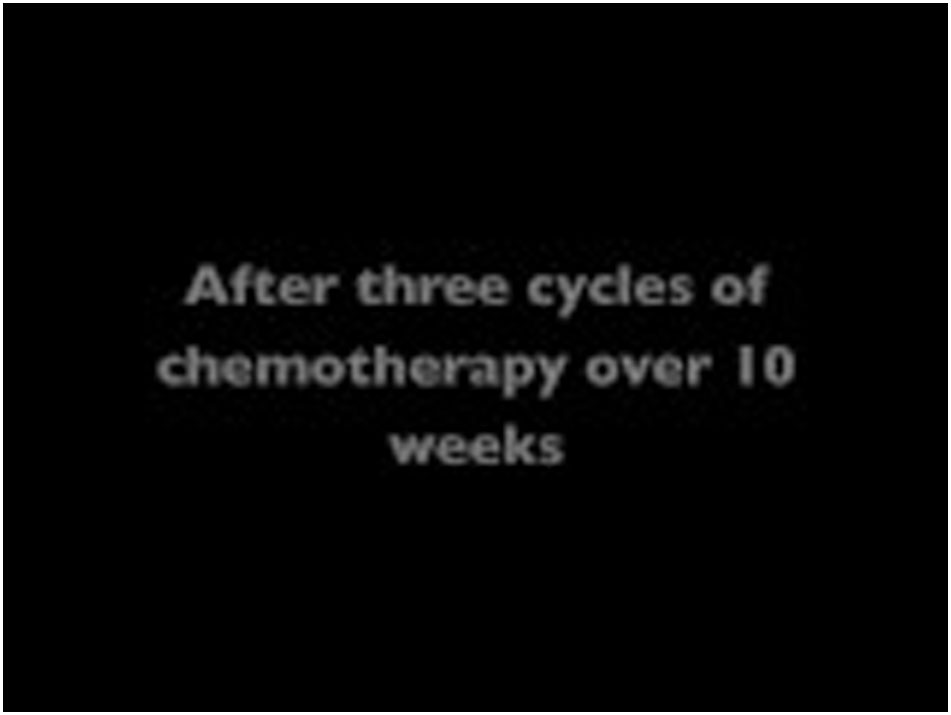

Supplement: Supplementary video — A video showing the laparoscopy finding for the sites that were sampled at presentation for whole genome sequencing prior to chemotherapy and the findings following the administration of three cycles of chemotherapy. Note the complete macroscopic resolution of the tumors. [file mmc13.jpg]
